# The Effects of Mixed *Robinia pseudoacacia* and *Quercus variabilis* Plantation on Soil Bacterial Community Structure and Nitrogen-Cycling Gene Abundance in the Southern Taihang Mountain Foothills

**DOI:** 10.3390/microorganisms12091773

**Published:** 2024-08-27

**Authors:** Yi Yang, Jing Chen, Yiwei Zheng, Rui Jiang, Yuqiang Sang, Jinsong Zhang

**Affiliations:** 1 College of Forestry, Henan Agricultural University, Zhengzhou 450046, China; yangyi023@henau.edu.cn (Y.Y.); m15538127056@163.com (J.C.); zhengyiwei_xyb@163.com (Y.Z.); jr159357159357@163.com (R.J.); 2 Henan Xiaolangdi Forest System National Research Station, Jiyuan 459000, China; zhangjs@caf.ac.cn; 3 Key Laboratory of Tree Breeding and Cultivation of State Forestry Administration, Research Institute of Forestry, Chinese Academy of Forestry, Beijing 100091, China

**Keywords:** high-throughput sequencing, pure forest, mixed forest, soil bacterial community, nitrogen-cycling functional genes

## Abstract

Mixed forests often increase their stability and species richness in comparison to pure stands. However, a comprehensive understanding of the effects of mixed forests on soil properties, bacterial community diversity, and soil nitrogen cycling remains elusive. This study investigated soil samples from pure *Robinia pseudoacacia* stands, pure *Quercus variabilis* stands, and mixed stands of both species in the southern foothills of the Taihang Mountains. Utilizing high-throughput sequencing and real-time fluorescence quantitative PCR, this study analyzed the bacterial community structure and the abundance of nitrogen-cycling functional genes within soils from different stands. The results demonstrated that Proteobacteria, Acidobacteria, and Actinobacteria were the dominant bacterial groups across all three forest soil types. The mixed-forest soil exhibited a higher relative abundance of Firmicutes and Bacteroidetes, while Nitrospirae and Crenarchaeota were most abundant in the pure *R*. *pseudoacacia* stand soils. Employing FAPROTAX for predictive bacterial function analysis in various soil layers, this study found that nitrogen-cycling processes such as nitrification and denitrification were most prominent in pure *R*. *pseudoacacia* soils. Whether in surface or deeper soil layers, the abundance of AOB *amoA*, *nirS*, and *nirK* genes was typically highest in pure *R*. *pseudoacacia* stand soils. In conclusion, the mixed forest of *R*. *pseudoacacia* and *Q*. *variabilis* can moderate the intensity of nitrification and denitrification processes, consequently reducing soil nitrogen loss.

## 1. Introduction

Bacteria, the most abundant and widely dispersed microorganisms in soil, are instrumental in vital soil processes such as organic matter decomposition and mineralization [1,2]. Emphasis on the diversity of soil bacterial communities is paramount in assessing soil quality. A higher bacterial diversity typically aligns with augmented soil resilience and resistance to stress [3,4]. A well-organized soil bacterial community can enhance the soil’s physical and chemical attributes, thereby boosting its fertility and promoting plant growth within forest ecosystems [5,6]. Conversely, soil properties may reciprocally affect the bacterial community structure. Moreover, the categorization of forest stands significantly influences the architecture of soil bacterial communities [7,8]. An analysis of the structure and diversity of bacterial communities in forest soils contributes to a more profound understanding of the complex relationship among soils, forests, and bacteria and fosters the healthy development of forest ecosystems.

The contrast between pure stands and mixed stands and their effect on soil bacterial communities have remained a central concern in soil ecology research. Compared to monospecific stands, mixed forests are typically more proficient in enhancing the chemical and physical properties of soil, thereby fostering tree growth and productivity [9]. The intermingling of different forest types inherently modifies vegetation. Such changes predominantly affect soil bacterial communities either directly or indirectly by transforming various soil characteristics, including pH, organic matter content, or the C/N ratio, as well as the quality and quantity of litterfall and root exudates [10,11,12,13]. Research has determined that specific bacterial taxa may be associated with different vegetation types [14,15,16]. Various vegetation types discharge disparate types and amounts of organic substances, such as root exudates and litterfall. These organic substances, laden with essential nutrients such as carbon, nitrogen, and phosphorus, are fundamental to bacterial growth and metabolism. The diverse nutrient compositions released by different vegetation types, therefore, create distinct bacterial communities in the soil. Mixed forests, characterized by richer species diversity and tree composition than pure stands, provide a wider range of root exudates and increased organic material input. As a result, mixed forests often display enhanced bacterial richness and diversity. Research illustrated a noteworthy correlation between forest biodiversity and species diversity [17]. In mixed forests teeming with varied vegetation types, the greater diversity of litter broadens the niches available for soil microorganisms. Other investigations propose that high plant diversity can cause an increase in litter and rhizodeposition, culminating in microbial community variations attributable to tree species diversity [18,19]. Moreover, in comparison to pure stands, mixed forests have the potential to raise soil pH, organic matter content, and nutrient availability, elements vital for the ecological adaptability and dispersion of soil bacteria [20]. The interplay between pure and mixed forests and soil bacterial communities is a nuanced process affected by a multitude of interacting factors, such as geographical location, soil type, climatic conditions, and management practices. Continuous experimental and analytical studies across diverse regions and ecosystems are thus essential for an exhaustive comprehension of the mechanisms that govern the influence of pure and mixed forests on soil bacterial communities.

The nitrogen cycle within the soil represents an indispensable facet of terrestrial ecosystem biogeochemical cycling and is recognized for its complex nature [21]. This cycle is instrumental in soil productivity, sustainability, and wider global environmental alterations [22]. Nitrification, a prominent phase of this cycle, entails the conversion of ammonia into nitrate through the processes of ammonia oxidation and nitrite oxidation [23,24]. Ammonia oxidation, catalyzed by ammonia monooxygenase (encoded by the *amoA* gene), is the rate-limiting stage in nitrification and involves microorganisms such as ammonia-oxidizing archaea (AOA) and ammonia-oxidizing bacteria (AOB) [23,25,26]. The regulation of nitrogen bioavailability in soil by these microorganisms reveals insights into nitrogen uptake by organisms and potential environmental nitrogen losses [27,28]. The diversity and functional characteristics of the nitrifying bacterial community have significant repercussions on nitrate production and nitrogen losses. Denitrification, another essential operation within the soil nitrogen cycle, converts nitrates into gaseous nitrogen oxides (such as nitric oxide and nitrous oxide) under anaerobic conditions [29]. This constitutes a primary avenue for greenhouse gas generation and facilitates nitrogen losses from the soil [30,31]. The denitrification process occurs in two stages: the reduction of nitrate to nitrite by nitrate reductase proteins, followed by the transformation of nitrite into gaseous nitrogen oxides through nitrite reductase proteins, encoded by the *nirS* and *nirK* genes [32]. These genes are often employed as molecular indicators for investigating the diversity and abundance of denitrifying bacteria. The nitrogen cycle in forest ecosystems is influenced by various factors, including vegetation type [33], microclimate [34], temperature [35,36], nitrogen deposition, litter decomposition, and disturbances such as fires. For example, research by Boyle et al. [37] determined that the nitrification potential in red alder trees in Oregon, USA, is markedly higher than that in Douglas fir. Transition zones from Andrus forests to grasslands exhibit significantly increased potentials for both nitrification and denitrification, with shifts in the bacterial communities involved in these processes [38,39,40]. Changes in vegetation type may incite alterations in the soil bacterial community, thus affecting the soil nitrogen cycle. Studies, such as those conducted by Rachid et al. [41], demonstrate that mixed forests encourage distinct microbial communities with a positive influence on soil nitrate concentrations. Other research also supports the notion that mixed forests augment soil nitrate content and overall nitrogen reserves [41,42,43,44,45,46]. Within mixed forests, the composition and diversity of tree species can affect the abundance and diversity of nitrifying bacteria and modulate the community composition and activity of denitrifying bacteria [47,48]. The profound interdependence between the soil nitrogen cycle and bacterial communities is well established. Bacterial communities, by manipulating soil nitrogen transformation processes and nitrogen use efficacy, play a vital role in orchestrating the soil nitrogen cycle. Soil properties exert a substantial effect on the structure and function of bacterial communities, affecting the efficiency and stability of the nitrogen cycle through the modulation of community composition and abundance. Past research has often focused narrowly on soil properties or bacterial communities, leading to gaps in understanding the mechanisms affecting nitrogen-cycling functions. Holistic investigations encompassing soil properties, bacterial community diversity, and nitrogen-cycling functional genes will illuminate the mechanisms controlling the soil nitrogen cycle, bearing important practical implications.

The southern foothills of the Taihang Mountains constitute a vital ecological bulwark in China. This region harbors native tree species such as *Robinia pseudoacacia* and *Quercus variabilis*, which are inherently connected to the local ecological milieu. *R. pseudoacacia* is valued mainly for its wood, soil improvement function, and nectar plant characteristics. *Q. variabilis* plays an important role in the economic and ecological fields due to its cork extraction and wood utilization value. Both have demonstrated their economic potential and environmental benefits in different fields. Although many studies have explored soil physicochemical properties, forest structure, and plant diversity within *R*. *pseudoacacia* mixed forests [49,50,51,52,53,54], there is a dearth of research on bacterial community structure and nitrogen-cycling functional genes. Based on the above research background, this study proposed the following scientific hypotheses: (1) Mixed forests changed the structure of soil bacterial communities, thereby improving the nitrogen cycle metabolism process, and (2) mixed forests improved the nitrogen retention capacity of the soil. Therefore, this study focused on the soils of mixed *R*. *pseudoacacia* and *Q*. *variabilis* forests in the low-mountain hilly terrain of the southern Taihang Mountains and used the soils of pure *R*. *pseudoacacia* forests and pure *Q*. *variabilis* forests as controls. By utilizing high-throughput sequencing methods and real-time quantitative PCR, we intend to contrast and analyze variations in soil bacterial community structures and the abundance of nitrogen-cycling functional genes between pure and mixed forests. Such inquiries hold significant value in improving the region’s nitrogen retention capacity and sustainable forestry practices, and our results will furnish a scientific foundation for the conservation of forest ecosystems and the formulation of soil management strategies.

## 2. Materials and Methods

### 2.1. Study Area Description

The designated study area resides within the Henan Xiaolangdi Forest System National Research Station (35°01′45″ N, 112°28′08″ E). Positioned in the characteristic low-mountain hilly region of the southern foothills of the Taihang Mountains, the location has an average altitude of 410 m. The soil typology in this region is characterized by relatively low water retention, mainly comprising brown soils and chernozems. This locale is integrated within the Chinese Forest Ecosystem Research Network (CFERN), and it predominantly hosts forest types such as *Q*. *variabilis*, *Platycladus orientalis*, and *R*. *pseudoacacia*. The understory vegetation largely consists of *Vitex negundo* and *Ziziphus jujuba*.

### 2.2. Experimental Design

Considering the conditions of artificially cultivated forests and their distribution, the typical vegetation restoration zone of the H Henan Xiaolangdi Forest System National Research Station was chosen for study (Figure S1). Research plots consisting of well-preserved forests of *R*. *pseudoacacia*, *Q*. *variabilis*, and mixed forests of both species were selected. These plots are uniform in topography and site conditions. Within each forest plot, six representative subplots of 2 m × 2 m were established, spaced 10 m apart. Surface vegetation or coverings were removed from each subplot. Using a five-point sampling method, soil samples from depths of 0–5 cm and 5–10 cm were collected with a soil auger. After removing plant residues, pebbles, and soil fauna, samples were sealed in self-sealing bags and transported to the laboratory. A portion of the soil samples was air-dried, crushed, and sieved in a shaded area for the determination of soil pH, organic matter, total nitrogen, available nitrogen, and available potassium. Another portion was stored at 4 °C for determining soil moisture content, nitrate nitrogen, ammonium nitrogen, soil microbial biomass carbon and nitrogen, nitrification potential, and denitrification potential. The remaining samples were stored at −20 °C for the extraction of soil DNA and the determination of gene abundance. Soil sampling was conducted in July 2021, collecting 6 samples from each layer within each forest type, totaling 36 samples.

### 2.3. Determination of Soil Physicochemical Properties

pH was measured using the potentiometric method (water–soil ratio of 2.5:1). The moisture content was determined using the oven-drying method. Organic matter was determined using the potassium dichromate titration method with external heating [55]. Nitrate and ammonium nitrogen were determined using the potassium chloride extraction method [56]. Total nitrogen was measured using the Kjeldahl method [57]. Alkali-hydrolyzable nitrogen was measured using the alkaline diffusion method [58]. Available phosphorus was determined using the sodium bicarbonate extraction–molybdenum antimony colorimetric method [59]. Available potassium was measured using the ammonium acetate extraction-flame photometry method [60]. Microbial biomass carbon and microbial biomass nitrogen were determined using the chloroform fumigation method [61,62]. Nitrification potential was measured based on the method reported by Yao et al. [63]. Denitrification potential was measured using the acetylene inhibition technique [64].

### 2.4. Soil DNA Extraction and Sequencing

Total DNA was extracted using a kit (OMEGA Bio Tek, Inc., Norcross, GA, USA) following the manufacturer’s instructions. The concentration and purity of the extracted DNA were checked using a NanoDrop2000 (Thermo Scientific, Waltham, MA, USA), while the quality of DNA extraction was verified using 1% agarose gel electrophoresis. Soil bacterial diversity was examined by amplifying the V3-V4 variable region of the 16S rRNA using the primers 341F: CCTAYGGGRBGCASCAG and 806R: GGACTACNNGGGTATCTAAT. PCR products were run on a 2% agarose gel and quantified using Quanti Fluor TM-ST (Promega, Madison, WI, USA). Purified amplicons were prepared for library construction according to the Illumina MiSeq platform (Illumina, San Diego, CA, USA) standard operating procedure. The constructed libraries were sequenced on the Illumina MiSeqPE300 platform to obtain raw data. To ensure accurate and reliable results, raw sequencing sequences were quality-controlled using Trimmomatic 0.39 software, assembled with FLASH 8.0 software, and clustered with UPARSE software (http://drive5.com/uparse/, accessed on 10 February 2022) based on a similarity threshold of 97% to obtain operational taxonomic units (OTUs). Singleton sequences and chimeras were removed during the clustering process. Each sequence was taxonomically classified using RDPclassifier (http://rdp.cme.msu.edu/, accessed on 12 February 2022).

### 2.5. Real-Time Fluorescent Quantitative PCR Analysis

Real-time fluorescent quantitative PCR was conducted using the TB Green Premix Ex Taq II kit (Takara, Shiga, Japan) on the ABI 7500 Real-time PCR System (Thermo Fisher, Waltham, MA, USA) to quantify the abundance of AOB *amoA*, *nirK*, and *nirS* genes. The standard curve for the real-time quantitative PCR was generated using plasmids containing the AOB *amoA*, *nirK*, and *nirS* genes. The reaction system for the real-time fluorescent quantitative PCR was 20 μL, which included 10 μL TB Green, 0.8 μL of each upstream and downstream primer, 0.4 μL of the dye, 2 μL of DNA template, and 6 μL of ultrapure sterile water. For details on the primers and reaction conditions used in the real-time fluorescent quantitative PCR, please refer to Table 1.

### 2.6. Statistical Analysis

Statistical analysis was performed using SPSS 17.0. A one-way analysis of variance (ANOVA) was employed. Prior to the analysis, descriptive statistics and normality tests were conducted. Post hoc multiple comparisons were then carried out using the LSD method, with the significance level set at α = 0.05. All nutrient content characteristics were measured based on the dry weight of the soil.

## 3. Results

### 3.1. Physicochemical Properties of Soils from Different Forest Types

In an evaluation across distinct forest types, the soil originating from the pure R. *pseudoacacia* forest exhibited notably elevated levels of pH, soil moisture content, alkali-hydrolyzable nitrogen, nitrate nitrogen, and available potassium in comparison to both the pure *Q. variabilis* forest and the mixed forest for the same soil layer (*p* < 0.05) (Table 2). Conversely, the soil from the pure *Q. variabilis* forest recorded the minimum levels of soil organic matter, total nitrogen, nitrate nitrogen, and available phosphorus. Surface soil analyses revealed no substantial variation in ammonium nitrogen content across the three forest types. When contemplating different soil layers within an identical forest type, the concentrations of moisture, organic matter, total nitrogen, alkali-hydrolyzable nitrogen, ammonium nitrogen, nitrate nitrogen, available potassium, and available phosphorus all significantly declined with increasing soil depth (*p* < 0.05). In contrast, soil pH values displayed a significant augmentation with increasing depth of the soil strata (*p* < 0.05).

Within the same soil layer, microbial biomass carbon, microbial biomass nitrogen, nitrification potential, and denitrification potential were all appreciably higher in the pure *R*. *pseudoacacia* forest than in the pure *Q*. *variabilis* forest and the mixed forest (*p* < 0.05). The disparities between the pure *Q*. *variabilis* forest and the mixed forest remained insignificant, excluding the microbial biomass carbon in the 0–5 cm soil layer. Intriguingly, microbial biomass carbon in both the pure *R*. *pseudoacacia* and pure *Q*. *variabilis* forests exhibited a significant decline with incremental soil depth (*p* < 0.05), whereas in the mixed forest, it notably increased with depth (*p* < 0.05). Apart from the microbial biomass nitrogen in the mixed forest, the surface soils of both pure *R*. *pseudoacacia* and *Q*. *variabilis* forests, as well as the nitrification and denitrification potentials in the surface soils of all three forest types, demonstrated higher values in comparison to the corresponding subsoil.

### 3.2. Diversity of Bacterial Communities in Different Forest Soils

The diversity indices of different forest types and different soil depths were compared, and the results are shown in Table 3. For the same soil layer across different forest types, the Shannon, Simpson, Chao1, and ACE indices were the highest in the pure *R*. *pseudoacacia* forest. For the 0–5 cm soil layer, the Shannon, Simpson, Chao1, and ACE indices of the mixed forest were not significantly different from those of the pure *R*. *pseudoacacia* forest, but all the diversity indices of the pure *Q*. *variabilis* forest, except for Chao1, were significantly lower than those of the pure *R*. *pseudoacacia* forest (*p* < 0.05). For the 5–10 cm soil layer, the Shannon, Chao1, and ACE indices of the pure *Q*. *variabilis* forest were not significantly different from those of the pure *R*. *pseudoacacia* forest, except for the Simpson index (0.996), which was significantly lower than that of the pure *R*. *pseudoacacia* forest (0.998) (*p* < 0.05). The diversity indices (except for the Simpson index) of the mixed forest were significantly lower than those of the pure *R*. *pseudoacacia* forest and the pure *Q*. *variabilis* forest (*p* < 0.05). The Shannon and Simpson indices in the pure *R*. *pseudoacacia* forest increased with soil depth, while the Chao1 and ACE indices decreased, but the differences between the two layers were not significant. In the pure *Q*. *variabilis* forest, the Shannon, Simpson, Chao1, and ACE indices increased with soil depth, while those of the mixed forest decreased.

**Figure 1:**
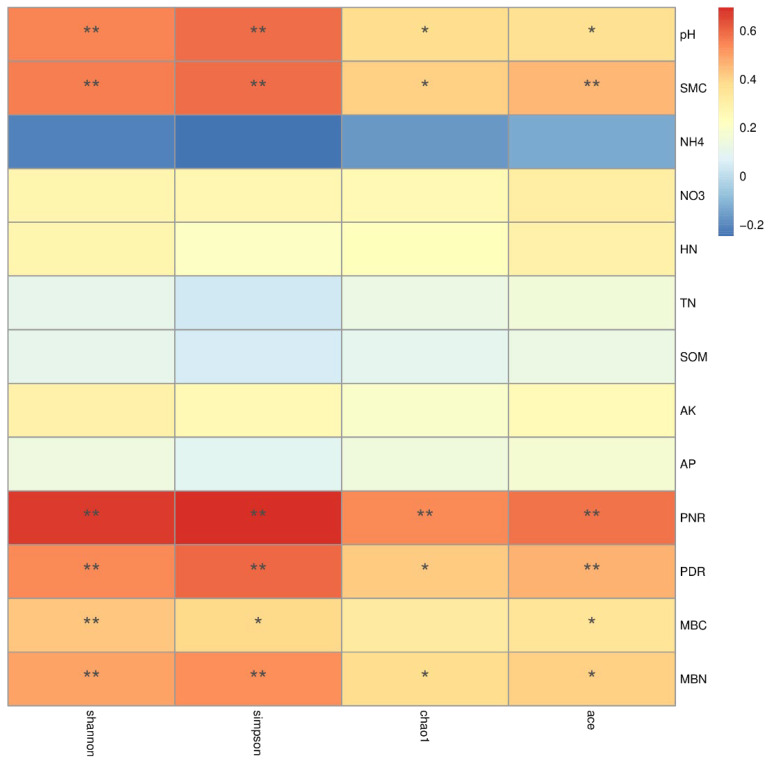
A Spearman analysis of bacterial community alpha diversity and environmental factors. *: significant correlation at the 0.05 level (two-sided); **: significant correlation at the 0.01 level (two-sided).

To further study the influence of environmental factors on the diversity of microbial communities in different forest soils, a Spearman analysis was conducted between environmental factors and diversity indices, as shown in Figure 1. pH, soil moisture content, nitrification potential, denitrification potential, microbial biomass carbon, and microbial biomass nitrogen all showed significant positive correlations with bacterial diversity (except for the correlation between microbial biomass carbon and Chao1) (*p* < 0.05). Additionally, nitrate nitrogen, alkali-hydrolyzable nitrogen, total nitrogen, organic matter, available potassium, and available phosphorus were also positively correlated with bacterial diversity. Among the measured physicochemical factors, only ammonium nitrogen was negatively correlated with bacterial diversity.

**Figure 2:**
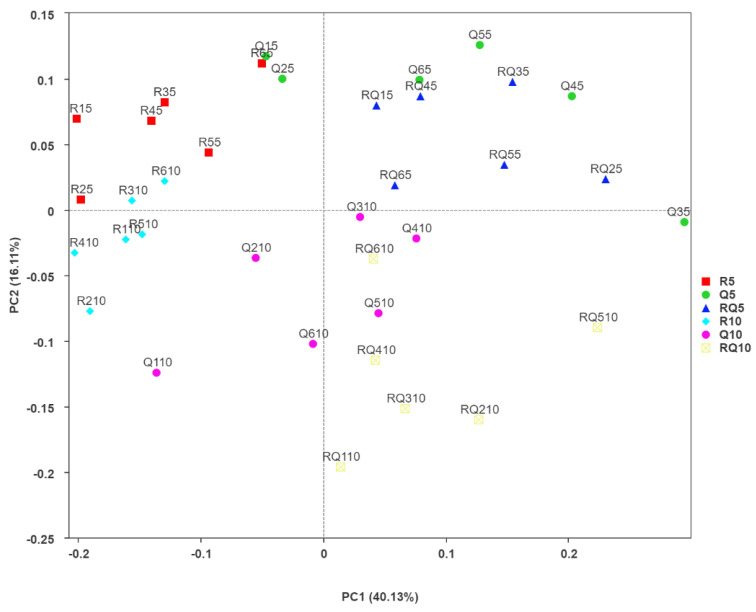
PCoA of bacterial community compositions based on the weighted UniFrac distance matrix.

A principal coordinate analysis (PCoA) based on weighted UniFrac distance was performed to compare the similarity of the bacterial community composition between different forest soil samples, as shown in Figure 2. The first and second coordinate axes explained 40.13% and 16.11% of the variability, respectively. The bacterial community composition differed significantly between the different soil layers of the same forest type (*p* < 0.05) (Table S1). For topsoil, there was no significant difference in bacterial community composition between the mixed forest and pure *Q*. *variabilis* forest, but there was significant difference between the pure *R*. *pseudoacacia* forest and pure *Q*. *variabilis* forest and mixed forest (ANOSIM, R = 0.6704, 0.8963, *p* < 0.05), as clearly separated in the PCoA plot. However, for the 5–10 cm soil layer, the differences in the composition of bacterial communities among the three forests were significant (*p* < 0.05) (Table S1).

**Figure 3:**
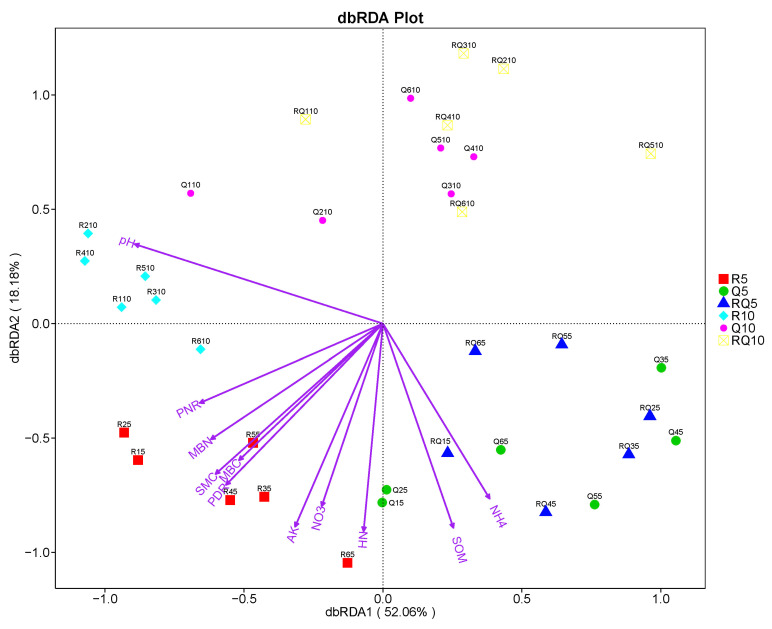
dbRDA analysis of bacterial community and environmental factors.

Investigating the effects of environmental factors on microbial community structure is one of the principal objectives of microbial ecology. This study organizes the data concerning bacterial community structures, fits the environmental factors, and analyzes the relationships between community composition and environmental factors. The microbial community composition matrix is subjected to detrended correspondence analysis (DCA), yielding a maximum gradient length (axis length) of 2.47, which is less than 3. Consequently, dbRDA analysis based on a linear model was chosen to explore the relationships between community structure and environmental factors. To minimize the influence of multicollinearity among environmental factors, those with variance inflation factors (VIF) greater than 20, such as total nitrogen and available phosphorus, were initially excluded. The dbRDA analysis was then applied to the remaining environmental factors to assess their effects on the bacterial community composition in various soil samples. The significance of these environmental factors was examined through the Mantel test. The results demonstrate that pH and nitrification potential are key environmental factors affecting the bacterial community structure (Table S2). The bacterial community composition in soil samples from pure *R*. *pseudoacacia* forests exhibited a significant positive correlation with pH and nitrification potential (*p* < 0.05), while soil samples from pure *Q*. *variabilis* forests and mixed forests showed a significant negative correlation with pH and nitrification potential (*p* < 0.05) (Figure 3).

### 3.3. Composition and Structure of Soil Bacterial Communities in Different Forest Stands

To further investigate the specific composition of bacterial communities in soil samples from various forest stands, comparative analyses were conducted at different taxonomic levels to assess the relative abundance differences in the dominant taxa among the soil layers of different forest stands.

**Figure 4:**
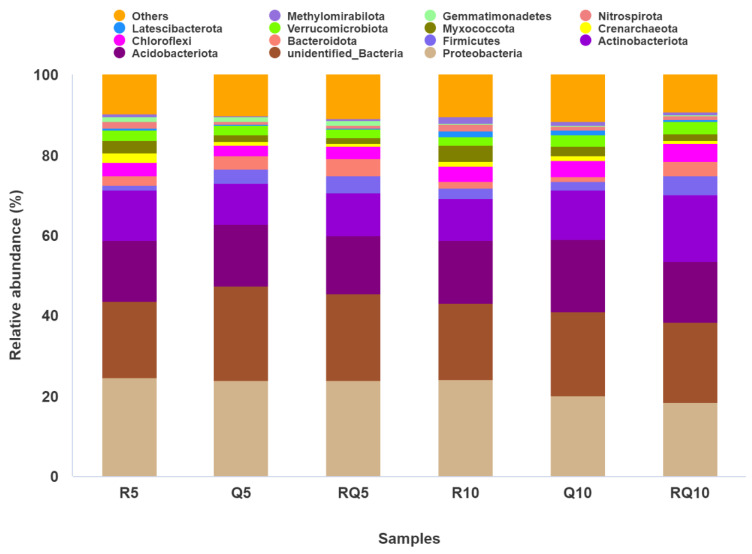
Relative abundance of dominant phyla in soil samples.

**Figure 5:**
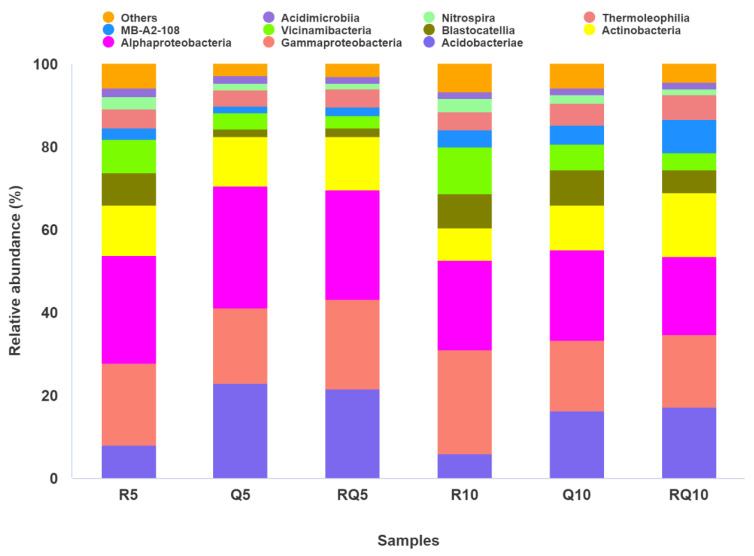
Relative abundance of dominant classes in abundant phyla.

At the phylum level, the top 14 abundant bacterial phyla were selected for analysis, yielding the following results (Figure 4). The bacterial phyla common to the two soil layers of the three forest stands included Proteobacteria, unidentified_Bacteria, Acidobacteria, Actinobacteria, Firmicutes, Bacteroidota, Chloroflexi, Crenarchaeota, Myxococcota, Verrucomicrobiota, Latescibacterota, Nitrospirota, etc. The relatively abundant bacterial phyla across all samples were mainly Proteobacteria, unidentified_Bacteria, Acidobacteria, and Actinobacteria. Among them, except for the slightly lower relative abundance of Proteobacteria in the subsoil of pure *Q*. *variabilis* (20.04%) and mixed-forest stands (18.56%) compared to unidentified_Bacteria (21.00%, 19.89%), Proteobacteria had the highest relative abundance in surface soil samples from three forest types and in subsoil samples from pure *R*. *pseudoacacia* stands. Furthermore, with the increase in soil layer depth within the same type of forest stand, the relative abundance of Proteobacteria decreased. Further analysis of Proteobacteria showed that α-Proteobacteria had the highest relative abundance in surface soil samples, followed by γ-Proteobacteria, with relative abundances of 25.96%, 29.31%, 26.64% and 19.78%, 18.35%, 21.50%, respectively, in the topsoil samples of pure *R*. *pseudoacacia* forest, pure *Q*. *variabilis* forest, and mixed forest (Figure 5). The relative abundance of α-Proteobacteria (21.83%, 18.89%) was also higher than that of γ-Proteobacteria (17.02%, 17.57%) in the subsoil samples of *Q*. *variabilis* and mixed forests, while the opposite was true in the subsoil of pure *R*. *pseudoacacia* stands (21.72% vs. 24.92%). Acidobacteria also had a high relative abundance, and its relative abundance increased with soil depth for the same stand type (Figure 4). Further analysis revealed that different samples had different dominant taxa (Figure 5). In the soil samples of pure *Q*. *variabilis* and mixed forests, the most abundant Acidobacteria was of Acidobacteriae, with relative abundances of 22.90% and 21.62% in surface soil and 16.41% and 17.20% in subsoil. In pure *R*. *pseudoacacia* forest soil samples, Vicinamibacteria was the most abundant within Acidobacteria, with relative abundances of 8.17% and 11.24% in the surface and subsoil, respectively. Blastocatellia had a lower relative abundance in the surface soil of *Q*. *variabilis* and mixed forests (1.71%, 2.12%) but a higher relative abundance in pure *R*. *pseudoacacia* surface soil and all three types of subsoil (5.42%–8.42%). For the topsoil, the relative abundance of Actinobacteria was the highest in pure *R*. *pseudoacacia* soil samples (12.51%), followed by mixed forest (10.66%) and pure *Q*. *variabilis* (10.16%), while for the subsoil, the relative abundance of Actinobacteria was the lowest in pure *R*. *pseudoacacia* (10.37%) and highest in mixed-forest samples (16.63%) (Figure 4). For Actinobacteria, the class Actinobacteria was the most dominant across all samples. The relative abundance of Actinobacteria was not significantly different in the surface soil samples of the three stands (11.99%–12.79%) but was significantly different in the subsoil samples (*t*-test, *p* < 0.05) (Figure 5). Compared to pure *R*. *pseudoacacia* and pure *Q*. *variabilis*, mixed-forest soils had a higher relative abundance of Firmicutes and Bacteroidetes, with 4.19% and 4.76% for Firmicutes and 4.33% and 3.69% for Bacteroidetes in the surface and subsoil, respectively. For the same type of forest stand, the relative abundance of Bacteroidetes decreased with soil depth, while that of Chloroflexi increased. Additionally, there were differences in the relative abundance of nitrogen-cycling-related Nitrospirae and Crenarchaeota among different forest soils. In all three types of forest stands, in both surface and subsoil samples, Nitrospirae and Crenarchaeota had the highest relative abundance in pure *R*. *pseudoacacia* and the lowest relative abundance in the mixed-forest sample.

**Figure 6:**
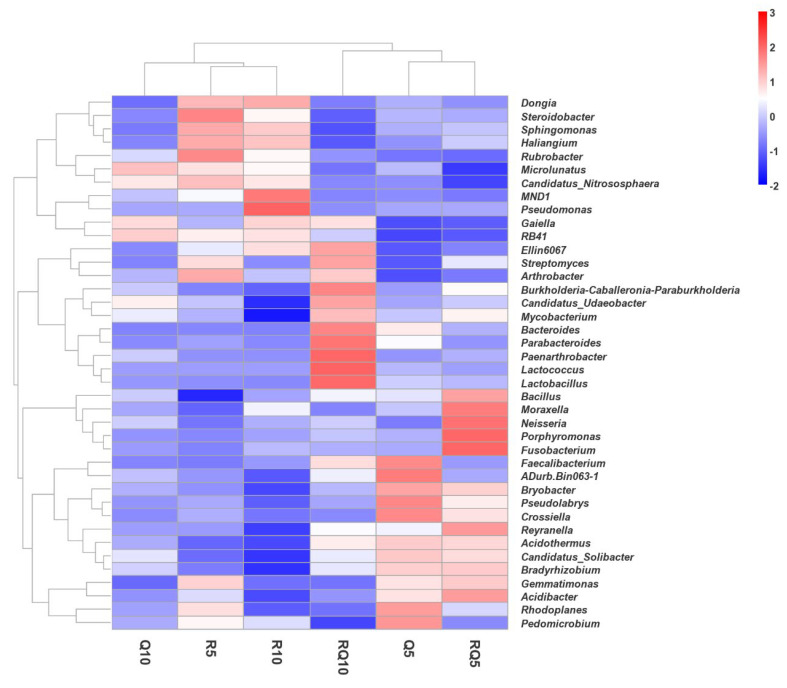
Heatmap analysis of relative abundance of dominant genera in soil samples.

Heatmaps were plotted for the top 40 genera across all samples, as shown in Figure 6. *Dongia*, *Steroidobacter*, *Sphingomonas*, *Haliangium*, *Rubrobacter*, and *Candidatus_Nitrososphaera* had higher relative abundances in pure *R*. *pseudoacacia* soil samples than in the pure *Q*. *variabilis* and mixed-forest soil samples. Except for *Dongia*, all other genera had higher relative abundance in surface soil samples of pure *R*. *pseudoacacia* compared to subsoil. *MND1* and *Pseudomonas* had the highest relative abundances in subsoil samples of pure *R*. *pseudoacacia*, at 2.36% and 0.43%, respectively. *Bacillus*, *Moraxella*, *Neisseria*, *Porphyromonas*, *Fusobacterium*, *Reyranella*, *Bradyrhizobium*, *Gemmatimonas*, and *Acidibacter* had the highest relative abundances in the mixed-forest surface soil samples. *Ellin6067*, *Streptomyces*, *Burkholderia-Caballeronia-Paraburkholderia*, *Candidatus_Udaeobacter*, *Mycobacterium*, *Bacteroides*, *Parabacteroides*, *Paenarthrobacter*, *Lactococcus*, and *Lactobacillus* had the highest relative abundances in the mixed-forest subsoil samples. *Faecalibacterium*, *ADurb.Bin063-1*, *Bryobacter*, *Pseudolabrys*, *Crossiella*, *Acidothermus*, *Candidatus_Solibacter*, *Rhodoplanes*, and *Pedomicrobium* had the highest relative abundances in the pure *Q*. *variabilis* surface soil samples.

**Figure 7:**
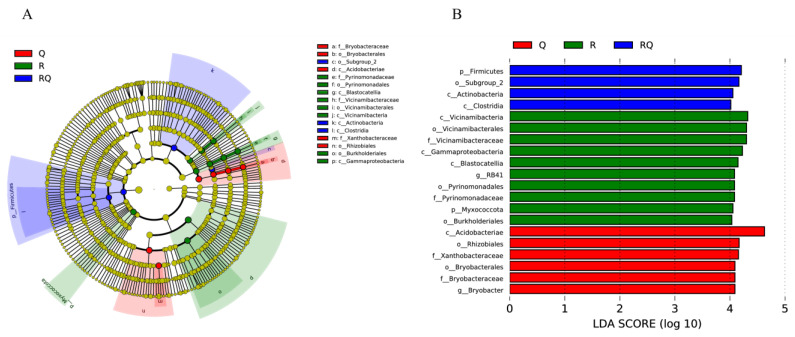
Linear discriminant analysis (LDA) effect size (LEfSe) analysis of relative bacterial abundance differences between soil samples in different stands. (**A**) Cladogram: green represents taxa with significantly higher relative abundance in pure *R*. *pseudoacacia* samples, red represents taxa with significantly higher relative abundance in pure *Q*. *variabilis* samples, blue represents taxa with significantly higher relative abundance in mixed *R*. *pseudoacacia* and *Q*. *variabilis* forest samples, and yellow represents no significant difference. The diameter of the circle reflects the relative abundance of that taxon. (**B**) LDA score histogram. Only taxa with LDA scores greater than 4.0 are marked in the figure.

To analyze the statistically significant microbial taxa among different forest stand types, LEfSe analysis was conducted on the identified bacteria at the phylum level, as shown in Figure 7. Vicinamibacteria, Vicinamibacterales, Vicinamibacteraceae, Gammaproteobacteria, Blastocatellia, RB41, Pyrinomonadaceae, Myxococcota, and Burkholderiales were significantly enriched in pure *R*. *pseudoacacia* soil samples. Acidobacteria, Rhizobiales, Xanthobacteraceae, Bryobacterales, Bryobacteraceae, and Bryobacter were significantly enriched in pure *Q*. *variabilis* soil samples. Firmicutes, Subgroup_2, Actinobacteria, and Clostridia were significantly enriched in mixed forest soil samples.

### 3.4. Ecological Function Prediction of Bacterial Communities in Different Forest Types

**Figure 8:**
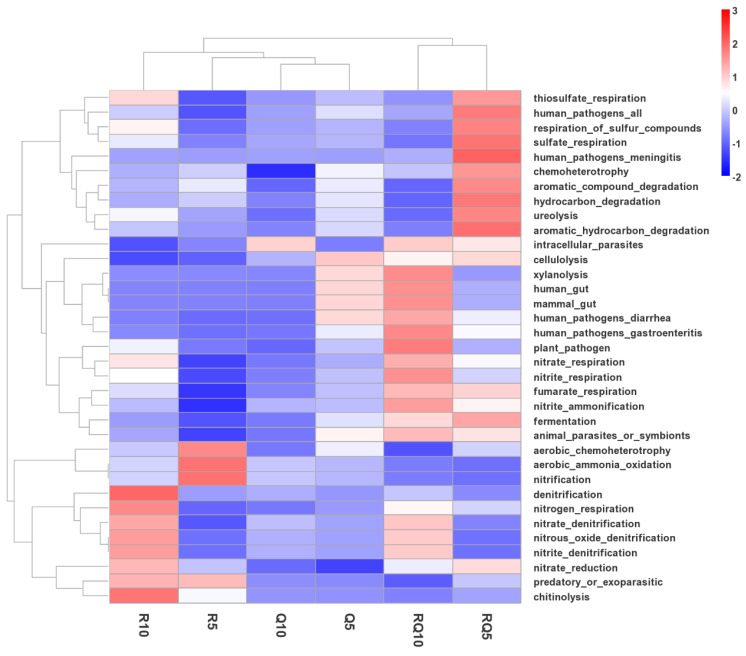
Heatmap analysis of relative abundance of dominant genera in soil samples. Prediction of microbial functions in different stands based on FAPROTAX.

Based on the Functional Annotation of Prokaryotic Taxa (FAPROTAX), the functionalities of bacterial communities in different soil layers of three forest types were predicted and analyzed. The results indicate that the relative abundances of functions associated with the nitrogen-cycling process, such as denitrification, nitrate denitrification, nitrous oxide denitrification, nitrite denitrification, and nitrate reduction, were highest in the lower soil samples of pure *R*. *pseudoacacia* forest. The relative abundances of aerobic ammonia oxidation and nitrification were highest in the surface soil samples of pure *R*. *pseudoacacia* forest (Figure 8). The relative abundances of nitrite ammonification, nitrite respiration, and nitrate respiration were highest in the lower soil samples of the mixed forest. Apart from the functions related to the nitrogen-cycling process, the relative abundances of plant pathogens, human pathogens, mammal gut, human gut, and xylanolysis were also highest in the lower soil samples of mixed forest. The relative abundances of fermentation, hydrocarbon degradation, aromatic hydrocarbon degradation, aromatic compound degradation, ureolysis, and chemoheterotrophy were highest in the surface soil samples of the mixed forest.

### 3.5. Abundance of Nitrogen-Cycling Functional Genes in Different Forest Types

**Figure 9:**
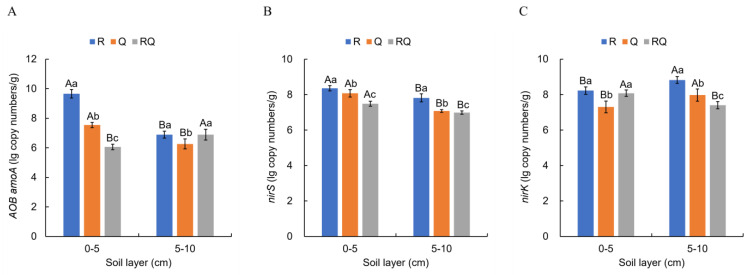
Abundance of nitrogen-cycling functional genes in different stands. (**A**) AOB *amoA* gene; (**B**) *nirS* gene; (**C**) *nirK* gene.

The abundance of nitrogen-cycling functional genes (AOB *amoA*, *nirS*, and *nirK*) in different soil layers of three forest types were analyzed. To make the data conform to a normal distribution, the gene copy numbers were logarithmically transformed as shown in Figure 9. The abundances of the AOB *amoA* gene in pure *R*. *pseudoacacia* forest, *Q*. *variabilis* forest, and mixed-forest surface soil samples were 9.66, 7.54, and 6.06 lg copy numbers·g^−1^, respectively, with significant differences among the three forest types (*p* < 0.05). In the lower soil samples, the abundances were 6.89, 6.26, and 6.89 lg copy numbers·g^−1^, respectively, with the *Q*. *variabilis* forest showing a significantly lower abundance of the AOB *amoA* gene compared to the pure *R*. *pseudoacacia* forest and mixed forest (*p* < 0.05). For both pure *R*. *pseudoacacia* and *Q*. *variabilis* forests, the abundance of the AOB *amoA* gene was significantly higher in the surface soil than in the lower soil (*p* < 0.05), while in the mixed forest, the surface soil displayed a significantly lower abundance than the lower soil (*p* < 0.05).

The abundances of the *nirS* gene in the pure *R*. *pseudoacacia* forest, *Q*. *variabilis* forest, and mixed-forest surface soil samples were 8.36, 8.07, and 7.49 lg copy numbers·g^−1^, respectively, with significant differences among the three forest types (*p* < 0.05). In the lower soil samples, the abundances were 7.81, 7.08, and 6.99 lg copy numbers·g^−1^, with a significant variance among the three forests (*p* < 0.05). Within the same forest type, the abundance of the *nirS* gene significantly decreased with increasing soil layer depth (*p* < 0.05).

The abundances of the *nirK* gene in the pure *R*. *pseudoacacia* forest, *Q*. *variabilis* forest, and mixed-forest surface soil samples were 8.23, 7.30, and 8.08 lg copy numbers·g^−1^, respectively, with the *Q*. *variabilis* forest having a significantly lower abundance of the *nirK* gene than the pure *R*. *pseudoacacia* and mixed forests (*p* < 0.05). In the lower soil samples, the abundances were 8.82, 7.98, and 7.40 lg copy numbers·g^−1^, with significant differences among the three forests (*p* < 0.05). In both pure *R*. *pseudoacacia* and *Q*. *variabilis* forests, the abundance of the *nirK* gene was significantly lower in the surface soil than in the lower soil (*p* < 0.05), whereas in the mixed forest, the surface soil had a significantly higher abundance than the lower soil (*p* < 0.05).

## 4. Discussion

### 4.1. Physicochemical Properties of Soil in Different Forest Stands

This study discovered that the soil moisture content in pure *R*. *pseudoacacia* stands was significantly higher than that in pure *Q*. *variabilis* stands and mixed stands. The reason that the soil moisture content in *R*. *pseudoacacia* is higher than that in the other two forest stands might be that the *Q*. *variabilis* leaves are larger and decompose more slowly than the *R*. *pseudoacacia* leaves. This forms a barrier on the forest floor, hindering some of the rainwater infiltration. Total nitrogen content is one of the main indicators of soil fertility, representing the total storage and potential supply of soil nitrogen [65,66]. Alkali-hydrolyzable nitrogen, including inorganic nitrogen and organic nitrogen directly available for plant absorption, can reflect the nitrogen nutritional status of plants [67]. The results of this study show that, for surface soil samples, the organic matter and total nitrogen content of mixed-forest soil are overall higher than those of pure *R*. *pseudoacacia* and *Q*. *variabilis* stands. This may be partly because the soil moisture content of *Q*. *variabilis* is low, and microbial activity is not high, leading to low organic matter and total nitrogen content. On the other hand, *R*. *pseudoacacia* root symbiotic nitrogen fixation is the main source of rhizosphere soil nitrogen, and *R*. *pseudoacacia* leaves are also rich in nitrogen elements. The input of leaf litter components and symbiotic nitrogen fixation together elevate the soil nitrogen content of *R*. *pseudoacacia* artificial forest ecosystems [68,69]. Among the three types of forest soils, the mixed forest has a slight advantage in total nitrogen content but is generally slightly lower in alkali-hydrolyzable nitrogen content than the pure *R*. *pseudoacacia* stand, suggesting that the mixture of *Q*. *variabilis* and *R*. *pseudoacacia* can enhance its soil nitrogen supply capacity.

The soil nitrification rate is closely related to pH [70] because under acidic conditions, NH_3_ readily combines with hydrogen ions to form inorganic ammonium nitrogen. Existing research has shown that ammonia molecules, not ammonium ions, are substrates for ammonia-oxidizing microorganisms [71]. Therefore, in acidic soil, ammonia-oxidizing microorganisms are in a substrate-deficient state, and soil nitrification intensity is lower; in addition, low pH can inhibit ammonia-oxidizing bacterial activity [72]. In this study, the pH of pure *Q*. *variabilis* stands and mixed stands was significantly lower than that of pure *R*. *pseudoacacia* stands (*p* < 0.05); thus, the soil of pure *R*. *pseudoacacia* stands is more conducive to nitrification. Moreover, the microbial biomass nitrogen content of pure *Q*. *variabilis* stands and mixed stands was significantly lower than that of pure *R*. *pseudoacacia* stands (Table 2), and the insufficient nitrogen supply for the microbes themselves might also be an important reason for the low potential for soil nitrification. pH is also an important factor affecting soil denitrification potential, and research by Zhang et al. [73] found that soil denitrification potential is significantly positively correlated with pH. Nitrate nitrogen is another key factor affecting soil denitrification potential, as it is both the substrate for denitrification and the electron acceptor for anaerobic respiration by denitrifying bacteria. Generally, the higher the nitrate nitrogen concentration is, the faster the growth and reproduction of denitrifying bacteria and the greater the denitrification potential [74]. In this study, the pH and nitrate nitrogen content of pure *R*. *pseudoacacia* stands were both significantly higher than those of pure *Q*. *variabilis* stands and mixed stands (*p* < 0.05); thus, among the three types of forest stands, the denitrification potential of pure *R*. *pseudoacacia* stands was the highest.

Soil microbial biomass carbon and nitrogen can characterize the vigorous degree of soil material metabolism and can be used to evaluate soil quality, microbial community status, and functional changes. There are significant differences in soil microbial biomass carbon and nitrogen among different forest types [75]. In this study, the content of soil microbial biomass carbon and nitrogen in the three types of forest soils was the highest in pure *R*. *pseudoacacia* stands, significantly higher than that in the other two stands (*p* < 0.05). This might be because *R*. *pseudoacacia* litter decomposes quickly, leading to high soil microbial biomass and activity and high soil nutrients. On the other hand, the effective nutrient content (AK, AP, and HN) of pure *R*. *pseudoacacia* stands is high, directly providing nutrients that can be absorbed and utilized by soil microbes.

In the 0–10 cm soil layer, as the soil layer deepens, the pH continues to increase, which may be related to the production of organic acids from the decomposition of surface litter [76]. Except for pH, the physicochemical properties and biological characteristics of the soil all show a surface accumulation phenomenon. In the 0–10 cm soil layer, soil nutrient content gradually decreases with the depth of the soil layer. The main reason is that the soil nutrients gather on the surface after the decomposition of plant litter, and their decomposition releases nutrients, including organic matter, hydrolyzable nitrogen, and phosphorus, mostly in insoluble or fixed forms. This leads to the difficulty of soil nutrient settlement, thus showing the surface aggregation of soil nutrients [77,78].

### 4.2. Bacterial Community Diversity in Different Forest Stands

Plant communities are significant sources of organic nutrients and energy essential for the survival of soil bacteria and can influence soil bacterial diversity by affecting soil parameters such as moisture content, carbon–nitrogen ratios, and pH levels [79]. Mixed forests, a common type of managed forest, can improve forest community structure and enhance forest ecological service functions. They may generate unique changes in organic carbon, total nitrogen, total phosphorus, soil temperature, and soil moisture, which can affect soil microbial diversity [80,81]. Many studies have posited that mixed forests, compared to pure stands, usually increase environmental heterogeneity and provide diverse food for microbes, thereby enhancing soil microbial diversity [82,83]. However, Hooper et al. [84] discovered that some mixed litter could antagonize microbes, reducing microbial diversity, while Nielsen et al. [85] found no direct effect of mixed forests on soil microbial diversity. The community composition and diversity of soil microbes are crucial indicators reflecting the ecological function of soil microbial communities [86]. In this study, for the upper soil layer, there were no significant differences in Chao1, ACE, Shannon, and Simpson indices between mixed forests of *R*. *pseudoacacia* and *Q*. *variabilis* and their pure stands. For the lower soil layer, these indices were significantly lower in mixed forests than in pure stands of *R*. *pseudoacacia* and *Q*. *variabilis* (*p* < 0.05), excluding the Simpson index between pure *Q*. *variabilis* stands and mixed stands. This may be related to differences in vegetation type that caused variations in soil physicochemical properties, thereby affecting the soil bacterial environment, species, quantity, composition, and distribution [87].

The soil bacterial Chao1, ACE, Shannon, and Simpson indices were significantly positively correlated with soil pH in this study, consistent with the results of Hartman et al. [88], who found that microbial diversity decreases with declining soil pH in acidic soils (pH < 6.5). Soil pH is a critical factor affecting soil metabolism and bacterial community structure and diversity [89,90]. Litter decomposition, an essential part of forest ecosystems, can directly alter soil pH [91]. Plants can modify soil pH physicochemical properties by secreting various organic acids, sugars, and phenols through root activities, affecting microbial growth and reproduction [92,93]. In soil with a low pH value, a higher concentration of H^+^ ions will disrupt the permeability and stability of bacterial cell membranes [94], inhibiting the efficiency of bacterial utilization of soil nutrients, thereby affecting soil bacterial diversity [95]. Bacterial community growth is positively correlated with pH value, and this phenomenon does not vary with different soil types [96].

Soil moisture content influences nutrient diffusion and chemical transformation [97] and affects soil microbial community composition and ecological functions [98]. Studies have shown that soil moisture content is a limiting factor for plant growth in arid and semiarid ecosystems and has a substantial influence on soil bacterial diversity [99]. Research by Shen et al. [100] on the alpine tundra found that soil carbon and nitrogen were key environmental factors affecting the soil bacterial altitude gradient distribution and emphasized the close relationship between soil moisture content and soil bacterial richness and diversity. Soil moisture content directly or indirectly affects microbial community structure through its influence on oxygen concentration and nutrient availability [101]. Research by Miranda et al. [102] found that the influence of soil moisture content on microbial communities can be divided into two stages. When soil moisture is low (above 50% of the soil’s saturated water content), soil moisture enhances the metabolic activity of the microbial community. As soil moisture continues to increase (reaching saturation), soil aeration decreases, leading to a reduction in the soil’s O_2_ content, and the gas diffusion process is hindered, thereby limiting the activity of aerobic microorganisms. This study’s results show that the Chao1, ACE, Shannon, and Simpson indices are significantly positively correlated with soil moisture content (*p* < 0.05). The soil moisture content in the three types of forest stands ranged from 9.48% to 19.91%. As the soil moisture content increases within a specific range, the soil’s oxygen content becomes appropriate, and the availability of inorganic elements such as nitrogen, phosphorus, and potassium and organic matter increases, providing conditions for more microbes to grow and reproduce [103,104].

Soil bacterial diversity was positively correlated with nitrate nitrogen, alkali-hydrolyzable nitrogen, and total nitrogen content and highly significantly positively correlated with nitrification potential and denitrification potential (*p* < 0.01) (Chao1 index was significantly positively correlated with denitrification potential (*p* < 0.05)). This may be related to soil microbial functional groups involved in the nitrogen cycle. Soil nitrogen content is a limiting factor for biological growth. Many studies have shown that changes in soil nitrogen content can cause changes in soil microbial biomass, microbial activity, and community composition [105]. The study of Gu et al. [106] on tea gardens under different fertilization conditions for a long time indicated that soil nitrate nitrogen content was a key factor affecting microbial diversity and community structure and that nitrate nitrogen content was significantly positively correlated with diversity indices. Organic matter, available potassium, and available phosphorus are important indicators for evaluating soil fertility [107] and were positively correlated with soil bacterial diversity, which is consistent with previous studies [108]. In this study, soil bacterial diversity is positively correlated with microbial biomass carbon and nitrogen. This is because microbial biomass carbon and nitrogen are active components of soil organic matter and act as primary constituents of the soil microbial biomass, participating in various ecological processes such as nutrient cycling in terrestrial ecosystems, litter decomposition, and organic matter transformation [109]. They play a key role in the carbon and nitrogen-cycling processes within forest ecosystems. Microbial biomass carbon and nitrogen are also indispensable and vital biological indicators for evaluating soil quality and health and serve as significant sources of soil available nutrients [110].

In this study, bacterial diversity was negatively correlated with ammonium nitrogen, possibly because an increase in soil ammonium nitrogen may lead to soil acidification [111]. It is generally believed that soil bacterial communities have higher richness and diversity under neutral pH conditions [96]. The positive correlation between soil bacterial diversity and pH in this study provides evidence for this relationship.

### 4.3. Soil Bacterial Community Composition of Different Forest Types

Numerous studies have demonstrated that the major bacterial communities in the soil of different forest ecosystems exhibit similarities at the phylum level, mainly dominated by phyla such as Proteobacteria, Acidobacteria, Actinobacteria, Bacteroidetes, and Planctomycetes [112,113,114]. In this study, the three types of forest soils were dominated by Proteobacteria, Acidobacteria, and Actinobacteria. Research by Zhang et al. [115] on mixed-forest and pure-forest soil bacterial communities showed that eight bacterial phyla, including Acidobacteria, Proteobacteria, Verrucomicrobia, Chloroflexi, and Actinobacteria, were the dominant groups. Studies by Wei et al. [116] in Baotianman and Jianfengling forests revealed that Proteobacteria and Acidobacteria were the dominant groups, and the relative abundances of Actinobacteria, Planctomycetes, and Chloroflexi were also high. Janssen’s research found that Proteobacteria and Acidobacteria dominated in different geographical regions and soil types [117]. Studies by Zhang et al. [118] on the bacterial community structure of different vegetation types in the northern foothills of the Qinghai–Tibet Plateau showed that meadows, grasslands, and desert grasslands were all dominated by Actinobacteria and Acidobacteria. The present study results are consistent with previous research. It can be seen that the ecological breadth of Proteobacteria, Actinobacteria, and Acidobacteria is wide, and they have strong adaptability to the environment. Changes in vegetation type have little effect on their dominant position.

Proteobacteria are generally found in nutrient-rich surface soil [119] and are eutrophic microbes capable of degrading amino acids and carbohydrates [120], enhancing soil nitrogen-fixing ability. Studies have shown that the relative abundance of Proteobacteria is positively correlated with carbohydrate metabolism and amino acid metabolism [121]. Mushinski et al. [122] believe that in forest ecosystems, the carbon and nitrogen metabolism in the soil surface is greater than that in the bottom layer, and soil carbon and nitrogen content can drive the underground ecosystem by influencing the bacterial community structure [123]. The present study shows that the relative abundance of Proteobacteria decreases with soil depth, consistent with the research of Zhang et al. [121]. In this study, α-Proteobacteria and γ-Proteobacteria were the dominant classes of Proteobacteria. α-Proteobacteria include bacteria that engage in symbiotic relationships with plants (such as Rhizobium), indicating that this phylum plays a vital role in the absorption and utilization of soil nutrients in forests.

Acidobacteria are oligotrophic bacteria [124] that can degrade plant debris polymers in soil [125] and participate in processes such as single-carbon compound metabolism [126], iron cycling [127], and photosynthesis [128]. In this study, the nutrient content in the lower soil layers of the same forest type, including organic matter, total nitrogen, alkali-hydrolyzable nitrogen, ammonium nitrogen, nitrate nitrogen, available potassium, and available phosphorus, was significantly lower than that in the surface soil (Table 2). Hence, the relative abundance of Acidobacteria was higher in the nutrient-poor lower soil. Vicinamibacteria and Blastocatellia belong to Acidobacteria; Vicinamibacteria prefer anaerobic environments and often occur in low oxygen environments [129], and Blastocatellia’s relative abundance is positively correlated with soil pH and negatively correlated with carbon content and alkali-hydrolyzable nitrogen [119]. In this study, the relative abundance of Vicinamibacteria in the lower soil of the pure *R*. *pseudoacacia* forest was the highest and the lowest in the surface soil of mixed forests, possibly due to the higher soil moisture and relatively lower soil oxygen concentration in the *R*. *pseudoacacia* forest. Blastocatellia’s relative abundance in the lower soil samples was higher than that in the surface soil, and it was lower in the surface soil samples of pure *Q*. *variabilis* and mixed forests. This may be because the pH of the pure *R*. *pseudoacacia* forest was significantly higher than that of the pure *Q*. *variabilis* and mixed forests, and within the same forest type, the pH of the lower soil was significantly higher than that of the surface soil.

Actinobacteria are Gram-positive bacteria with the ability to degrade lignin and decompose cellulose [130]. They also participate in the nitrogen cycle, contributing to the nitrification and denitrification process in soil nitrogen elements [131]. Pure *R*. *pseudoacacia* forest, pure *Q*. *variabilis* forest, and mixed-forest surface and lower soil layers contain a higher abundance of Actinobacteria because Actinobacteria is a group of microbes that can adapt well to environmental stresses such as drought and high salt and survive in infertile soils [132].

The relative abundance of Firmicutes was the highest in mixed-forest samples, possibly suitable for growth in low nutrient environments. On the other hand, it is common for microorganisms to enter a dormant state under harsh environmental conditions, especially those capable of forming spores and sporangia. Bacteroidetes tend to accumulate in nutrient-rich environments [124] and play a significant role in the nitrogen cycle [133,134]. Therefore, for pure *R*. *pseudoacacia*, pure *Q*. *variabilis*, and mixed forests, the relative richness of Bacteroidetes in the surface soil is higher than that in the lower soil. Chloroflexi are bacteria that generate energy through photosynthesis without producing oxygen. They are facultatively anaerobic and suitable for surviving in arid and barren soils [124,135,136]. This study shows that the relative abundance of Chloroflexi in the lower soil is greater than that in the surface soil of the same forest type, possibly related to the lower water and organic matter content in the lower soil. Nitrospirae play an essential role in the oxidation process of nitrite [137]; in environments with high nitrogen content, the abundance of Nitrospirae is high [138], and its relative abundance promotes nitrification. Whether in surface soil or lower soil, Nitrospirae and Crenarchaeota involved in the nitrification process had the highest relative abundance in pure *R*. *pseudoacacia* forest samples, consistent with the highest nitrification potential of pure *R*. *pseudoacacia* forest samples. At the genus level, the Crenarchaeota bacteria *Candidatus Nitrososphaera* and Proteobacteria bacteria *MND1* also had the highest relative abundance in pure *R*. *pseudoacacia* forest samples. Previous research has shown that *Candidatus Nitrososphaera* is affected by the soil carbon-to-nitrogen ratio and pH value and is positively correlated with pH within a certain range [139]. The highest relative abundance of *Candidatus Nitrososphaera* in pure *R*. *pseudoacacia* forest samples may be related to its highest pH.

### 4.4. Nitrogen Cycle Functional Gene Abundance in Different Forest Types

Ammonia oxidation is a key link in the nitrogen cycle process, whereby NH_3_ is converted to NO_2_^-^ under the action of ammonia-oxidizing microorganisms and eventually to NO_3_^-^. The key enzyme that allows ammonia-oxidizing microorganisms to oxidize ammonia to hydroxylamine is ammonia monooxygenase. This enzyme is encoded by three genes: *amoA*, *amoB*, and *amoC* [140]. Typically, the *amoA* gene is utilized as a molecular marker to study the community structure of nitrifying bacteria or to assess the strength of nitrification. In this study, the abundance of *amoA* gene in *R*. *pseudoacacia* and *Q*. *variabilis* significantly decreased with increasing soil depth (*p* < 0.05) (Figure 9), consistent with existing research findings [141,142]. This trend may be primarily due to the higher content of nutrients such as organic matter, ammonium nitrogen, available potassium, and available phosphorus in the surface soil, which facilitates microbial access to sufficient nutrients and substrates. As soil depth increases and nutrient content gradually diminishes, microbial growth becomes constrained, and microbial quantities decrease. Additionally, ammonia oxidation generally occurs under aerobic conditions, and as soil depth increases, the oxygen concentration continually drops. Hence, for *R*. *pseudoacacia* and *Q*. *variabilis*, the abundance of *amoA* gene in surface soil is significantly greater than that in deeper soil (*p* < 0.05). FAPROTAX functional predictions indicate that the relative abundance of aerobic ammonia oxidation and nitrification is highest in surface soil samples of pure *R*. *pseudoacacia* forest (Figure 8), and therefore, the abundance of AOB *amoA* gene is also the highest there. For mixed-forest soil, the abundance of *amoA* gene in the deeper soil is greater than that in surface soil, possibly due to factors other than nutrients and oxygen, such as pH. pH strongly affects the physiological metabolism of ammonia-oxidizing bacteria [143]. In soil with lower pH, these bacteria are relatively fewer and less active, resulting in weaker nitrification. It is generally believed that ammonia-oxidizing bacteria do not grow easily in environments with pH < 6.5, instead having a suitable growth pH range of 7.0–8.5. The pH of the mixed-forest surface soil is 6.19 (Table 2), which is outside the optimal growth range for ammonia-oxidizing bacteria and may have resulted in a lower abundance of the AOB *amoA* gene.

The reduction of nitrite to NO is the most critical rate-limiting step in the denitrification process and is catalyzed by the nitrite reductase enzyme. This enzyme exists in two forms, a soluble copper-containing enzyme and a cytochrome enzyme, encoded by the *nirK* and *nirS* genes, respectively [144]. Although these two enzymes have the same function, they differ in structure and catalytic sites and typically do not coexist within the same denitrifying species, representing two different ecological types of denitrifiers occupying different niches [145]. Different soil physicochemical characteristics will result in various denitrifying microbial communities [146]. In this study, the *nirS* gene abundance in all three forest types significantly decreased with increasing soil depth (*p* < 0.05) (Figure 9), similar to findings in both forest [147] and agricultural soils [148]. This might be associated with the lower water content in deeper soil. For the same type of forest, the surface soil water content is significantly higher than that of deeper soil (Table 2). Soil moisture is an essential factor affecting soil nitrogen cycling. Soil water content and other physicochemical properties can significantly alter soil porosity, thereby affecting oxygen circulation in soil and, consequently, microbial activity [149,150]. Under varying moisture conditions, the abundance of the *nirS* gene in soil increases with increasing water content [151]. In mixed-forest soil, the *nirK* gene abundance significantly decreases with increasing soil depth (*p* < 0.05), whereas in pure *R*. *pseudoacacia* and *Q*. *variabilis* forests, the *nirK* gene abundance significantly increases with increasing soil depth (*p* < 0.05) (Figure 9). This might be due to the lower oxygen content in deep soil, promoting the growth of *nirK*-type denitrifying bacteria. Apart from mixed-forest surface soil samples, both *nirS* and *nirK* gene abundance in pure *R*. *pseudoacacia* forest soil is significantly greater than that in pure *Q*. *variabilis* and mixed-forest soil (*p* < 0.05), likely related to the highest abundance of the AOB *amoA* gene in pure *R*. *pseudoacacia* forest soil. Study by Ribbons et al. [152] has found a significant positive correlation between the total copy number of the AOA *amoA* and AOB *amoA* gene and the total copy number of the *nirK* and *nirS* genes. Ammonia-oxidizing microorganisms convert ammonium nitrogen to nitrate nitrogen through nitrification, providing substrates for denitrification and thus increasing denitrifying microbial gene abundance. In addition, the denitrification potential in pure *R*. *pseudoacacia* forest is significantly greater than that in pure *Q*. *variabilis* and mixed forest (*p* < 0.05) (Table 2). Research by Zhang et al. [153] has revealed that potential denitrification rates are closely related to the abundance of the *nirK* and *nirS* genes.

Nitrification is the process of oxidizing ammonia to nitrate, converting it from a cation to an anion. Since the colloidal particles in the surface layer of most soils are negatively charged, cations are easily adsorbed by soil particles, whereas anions are not easily adsorbed. Therefore, nitrate anions can easily be leached from soil, increasing nitrogen loss and impairing nitrogen retention. The denitrification process not only results in significant nitrogen fertilizer loss in forest soil but also releases the greenhouse gases NO and N_2_O, contributing to global warming [154]. Therefore, in forest soil ecosystems, efforts should be made to minimize nitrification and denitrification. From this perspective, among the three types of forest soils, mixed-forest soil has the lowest abundance of the AOB *amoA*, *nirS*, and *nirK* genes, and its nitrification potential and denitrification potential are also the lowest, thus favoring nitrogen retention in the soil.

This study demonstrates that mixed planting of *R*. *pseudoacacia* and *Q*. *variabilis* has significant effects in enhancing soil nutrients compared to planting pure *Q*. *variabilis* forest. Regarding nitrification and denitrification, it reduces the intensity of nitrification and denitrification to a certain extent, minimizing soil nitrogen loss. It is suggested that further improvements in soil nutrient conditions could be achieved by adjusting the mixing ratio and exploring planting patterns that maximize nitrogen retention and minimize nitrogen loss.

## 5. Conclusions

The results demonstrated that, excluding soil pH, the soil physicochemical properties exhibited surface aggregation. Proteobacteria, Acidobacteria, and Actinobacteria were the dominant bacterial groups across all three forest soil types. The mixed-forest soil exhibited a higher relative abundance of Firmicutes and Bacteroidetes, while Nitrospirae and Crenarchaeota were most abundant in the pure *R*. *pseudoacacia* stand soils. Under the same site conditions and management measures in the low mountainous and hilly areas of the southern Taihang Mountains, the nitrification potential, denitrification potential, and abundance of nitrification gene (AOB *amoA*) and denitrification genes (*nirS*, *nirK*) in the mixed forests of *R*. *pseudoacacia* and *Q*. *variabilis* are lower than those in pure *R*. *pseudoacacia* forests. Therefore, from the perspective of soil nitrogen retention, the construction of mixed forests of *R*. *pseudoacacia* and *Q*. *variabilis* in this region is superior to that of pure *R*. *pseudoacacia* forests. Subsequent studies need to analyze the impact of the planting ratio of mixed forests on soil properties and microbial community structure, so as to provide a theoretical basis for further exploring the optimal configuration model for artificial forests in the region.

## Figures and Tables

**Table 1:** Primers for real-time fluorescent quantitative PCR amplification of nitrogen-cycling functional genes.

Target Gene	Primer Name	Primer Sequence (5′-3′)	Real-Time Fluorescent Quantitative PCR Reaction Conditions
AOB amoA	amoA-1F	GGGGTTTCTACTGGTGGT	95 °C/30 s; 40 cycles of 95 °C/5 s, 58 °C/34 s
	amoA-2R	CCCCTCKGSAAAGCCTTCTTC	
nirS	Cd3AF	GTSAACGTSAAGGARACSGG	95 °C/30 s; 45 cycles of 95 °C/5 s, 60 °C/40 s
	R3cd	GASTTCGGRTGSGTCTTGA	
nirK	1F	GGMATGGTKCCSTGGCA	95 °C/30 s; 40 cycles of 95 °C/5 s, 60 °C/40 s
	5R	GCCTCGATCAGRTTRTGGTT	

**Table 2:** Physicochemical properties of soils in different stands.

Plantation	Soil Layer /(cm)	SMC /(%)	pH	SOM /(g/kg)	TN /(g/kg)	HN/ (mg/kg)	NH_4_^+^-N /(mg/kg)	NO_3_^-^-N /(mg/kg)	AK /(mg/kg)	AP /(mg/kg)	MBC /(mg/kg)	MBN /(mg/kg)	PNR /(mg/kg/d)	PDR /(mg/kg/d)
R	0–5	19.91 ± 3.65 Aa	7.04 ± 0.21 Ba	78.44 ± 17.54 Aab	5.24 ± 0.97 Aa	176.64 ± 17.16 Aa	12.27 ± 0.81 Aa	54.65 ± 15.44 Aa	210.57 ± 19.60 Aa	5.30 ± 0.92 Aa	298.61 ± 36.85 Aa	39.51 ± 9.28 Aa	12.07 ± 6.99 Aa	60.11 ± 9.58 Aa
	5–10	15.57 ± 1.75 Ba	7.80 ± 0.10 Aa	28.01 ± 9.10 Ba	2.13 ± 0.55 Ba	79.45 ± 13.94 Ba	9.75 ± 0.53 Bab	25.81 ± 5.19 Ba	132.92 ± 8.89 Ba	2.16 ± 0.52 Ba	126.04 ± 37.75 Ba	21.39 ± 2.99 Ba	6.16 ± 2.65 Aa	36.26 ± 8.46 Ba
Q	0–5	12.38 ± 1.03 Ab	6.50 ± 0.40 Bb	66.51 ± 15.66 Ab	4.11 ± 0.55 Ab	123.10 ± 17.70 Ab	12.55 ± 0.85 Aa	26.68 ± 3.74 Ab	137.69 ± 11.22 Ab	3.49 ± 0.17 Ab	138.45 ± 35.80 Ab	15.63 ± 1.51 Ab	0.76 ± 0.86 Ab	26.07 ± 7.13 Ab
	5–10	9.48 ± 0.81 Bb	7.32 ± 0.30 Ab	18.18 ± 3.73 Bb	1.61 ± 0.25 Ba	48.25 ± 3.41 Bb	9.61 ± 0.35 Bb	13.73 ± 1.04 Bb	57.62 ± 4.63 Bb	1.21 ± 0.16 Bb	82.54 ± 15.82 Bb	12.39 ± 3.95 Ab	0.05 ± 0.03 Ab	12.97 ± 2.44 Bb
RQ	0–5	12.43 ± 0.78 Ab	6.19 ± 0.42 Bb	91.58 ± 13.43 Aa	5.61 ± 0.69 Aa	140.10 ± 38.19 Ab	11.79 ± 0.84 Aa	36.19 ± 0.65 Ab	130.37 ± 15.30 Ab	4.91 ± 0.76 Aa	45.55 ± 4.36 Bc	9.30 ± 3.14 Ab	0.25 ± 0.13 Ab	27.30 ± 5.01 Ab
	5–10	10.16 ± 0.64 Bb	6.88 ± 0.37 Ac	26.37 ± 4.08 Bab	2.07 ± 0.28 Ba	44.70 ± 11.35 Bb	10.18 ± 0.65 Ba	15.39 ± 2.36 Bb	72.03 ± 12.08 Bc	2.25 ± 0.18 Ba	56.26 ± 8.15 Ab	9.86 ± 3.67 Ab	0.09 ± 0.07 Bb	15.27 ± 5.62 Bb

R: *R*. *pseudoacacia* plantation; Q: *Q*. *variabilis* plantation; RQ: Mixed plantation of *R*. *pseudoacacia* and *Q*. *variabilis*. SMC: Soil moisture content; SOM: Soil organic matter; TN: Total nitrogen; HN: Alkali-hydrolyzable nitrogen; NH_4_^+^-N: Ammonium nitrogen; NO_3_^-^-N: Nitrate nitrogen; AK: Available potassium; AP: Available phosphorus; MBC: Microbial biomass carbon; MBN: Microbial biomass nitrogen; PNR: Nitrification potential; PDR: Denitrification potential. The same applies below. Different capital letters indicate significant differences between soil layers in the same stand, *p* < 0.05; Different lowercase letters indicate significant differences between stand types in the same soil layer, *p* < 0.05.

**Table 3:** Alpha diversity of soil bacterial communities in different stands.

Sample	Shannon	Simpson	Chao1	ACE
R5	9.91 ± 0.09 Aa	0.997 ± 0.00 Ba	3641.70 ± 232.99 Aa	3753.28 ± 253.81 Aa
Q5	9.44 ± 0.47 Ab	0.995 ± 0.00 Ab	3079.28 ± 549.90 Aa	3139.68 ± 549.43 Ab
RQ5	9.59 ± 0.30 Aab	0.996 ± 0.00 Aab	3300.94 ± 409.77 Aa	3407.28 ± 424.25 Aab
R10	9.97 ± 0.23 Aa	0.998 ± 0.00 Aa	3585.26 ± 347.20 Aa	3673.87 ± 321.55 Aa
Q10	9.70 ± 0.29 Aa	0.996 ± 0.00 Ab	3562.25 ± 481.29 Aa	3591.92 ± 453.83 Aa
RQ10	9.23 ± 0.32 Ab	0.995 ± 0.00 Ab	2823.21 ± 507.57 Ab	2819.65 ± 489.59 Ab

R5, Q5, and RQ5 represent 0–5 cm soil samples of pure *R*. *pseudoacacia*, *Q*. *variabilis*, mixed *R*. *pseudoacacia,* and *Q*. *variabilis* forests, respectively; R10, Q10, and RQ10 represent 5–10 cm soil samples of pure *R*. *pseudoacacia*, *Q*. *variabilis*, mixed *R*. *pseudoacacia,* and *Q*. *variabilis* forests, respectively. The same applies below. Different capital letters indicate significant differences between soil layers in the same stand, *p* < 0.05; Different lowercase letters indicate significant differences between stand types in the same soil layer, *p* < 0.05.

## Data Availability

The raw data supporting the conclusions of this article will be made available by the authors on request.

## Supplementary Materials

The following supporting information can be downloaded at: https://www.mdpi.com/article/10.3390/microorganisms12091773/s1, Figure S1: Soil sampling site and scheme; Table S1: Similarity analysis of bacterial communities among different samples; Table S2: Correlation between bacterial community structure and soil environmental factors tested by Mantel.

## References

[1] Heijden M.G.A.V.D., Bardgett R.D., Straalen N.M.V. (2008). The unseen majority: Soil microbes as drivers of plant diversity and productivity in terrestrial ecosystems. Ecol. Lett..

[2] Comerford N.B., Franzluebbers A.J., Stromberger M.E., Morris L., Markewitz D., Moore R. (2013). Assessment and evaluation of soil ecosystem services. Soil Horiz..

[3] Kumawat K.C., Sharma B., Nagpal S., Kumar A., Tiwari S., Nair R.M. (2023). Plant growth-promoting rhizobacteria: Salt stress alleviators to improve crop productivity for sustainable agriculture development. Front. Plant Sci..

[4] Preece C., Verbruggen E., Liu L., Weedon J.T., Peñuelas J. (2019). Effects of past and current drought on the composition and diversity of soil microbial communities. Soil Biol. Biochem..

[5] Dincă L.C., Grenni P., Onet C., Onet A. (2022). Fertilization and Soil Microbial Community: A Review. Appl. Sci..

[6] Sofo A., Mininni A.N., Ricciuti P. (2020). Soil Macrofauna: A key Factor for Increasing Soil Fertility and Promoting Sustainable Soil Use in Fruit Orchard Agrosystems. Agronomy.

[7] Lladó S., López-Mondéjar R., Baldrian P. (2018). Drivers of microbial community structure in forest soils. Appl. Microbiol. Biotechnol..

[8] Nacke H., Goldmann K., Schöning I., Pfeiffer B., Kaiser K., Castillo-Villamizar G.A., Schrumpf M., Buscot F., Daniel R., Wubet T. (2016). Fine spatial scale variation of soil microbial communities under European Beech and Norway Spruce. Front. Microbiol..

[9] Pretzsch H., del Río M., Ammer Ch Avdagic A., Barbeito I., Bielak K., Brazaitis G., Coll L., Dirnberger G., Drössler L., Fabrika M. (2015). Growth and yield of mixed versus pure stands of Scots pine (*Pinus sylvestris* L.) and European beech (*Fagus sylvatica* L.) analysed along a productivity gradient through Europe. Eur. J. For. Res..

[10] Augusto L., De Schrijver A., Vesterdal L., Smolander A., Prescott C., Ranger J. (2015). Influences of evergreen gymnosperm and deciduous angiosperm tree species on the functioning of temperate and boreal forests. Biol. Rev. Camb. Philos. Soc..

[11] Jin X., Liu Y., Hu W., Wang G., Kong Z., Wu L., Ge G. (2019). Soil Bacterial and Fungal Communities and the Associated Nutrient Cycling Responses to Forest Conversion after Selective Logging in a Subtropical Forest of China. For. Ecol. Manag..

[12] Nie H., Qin T., Yan D., Lv X., Wang J., Huang Y., Lv Z., Liu S., Liu F. (2021). How do tree species characteristics affect the bacterial community structure of subtropical natural mixed forests?. Sci. Total Environ..

[13] Wan P., He R. (2020). Soil microbial community characteristics under different vegetation types at the national nature reserve of Xiaolongshan Mountains, Northwest China. Ecol. Inform..

[14] Aleklett K., Leff J.W., Fierer N., Hart M. (2015). Wild plant species growing closely connected in a subalpine meadow host distinct root-associated bacterial communities. PeerJ.

[15] Ling N., Wang T., Kuzyakov Y. (2022). Rhizosphere bacteriome structure and functions. Nat. Commun..

[16] Yao F., Yang S., Wang Z., Wang X., Ye J., Wang X., DeBruyn J.M., Feng X., Jiang Y., Li H. (2017). Microbial Taxa Distribution Is Associated with Ecological Trophic Cascades along an Elevation Gradient. Front. Microbiol..

[17] Benizri E., Amiaud B. (2005). Relationship between plants and soil microbial communities in fertilized grasslands. Soil Biol. Biochem..

[18] de Deyn G.B., van der P.W.H. (2005). Linking aboveground and belowground diversity. Trends Ecol. Evol..

[19] Kowalchuk G.A., Buma D.S., de Boer W., Klinkhamer P.G.L., van Veen J.A. (2002). Effects of above-ground plant species composition and diversity on the diversity of soil-borne microorganisms. Antonie Leeuwenhoek.

[20] Lladó S., López-Mondéjar R., Baldrian P. (2017). Forest Soil Bacteria: Diversity, Involvement in Ecosystem Processes, and Response to Global Change. Microbiol. Mol. Biol. Rev..

[21] Thamdrup B. (2012). New pathways and processes in the global nitrogen cycle. Annu. Rev. Ecol. Evol. Syst..

[22] Canfield D.E., Glazer A.N., Falkowski P.G. (2010). The evolution and future of Earth’s nitrogen cycle. Science.

[23] Hao J., Feng Y., Wang X., Yu Q., Zhang F., Yang G., Ren G., Han X., Wang X., Ren C. (2022). Soil microbial nitrogen-cycling gene abundances in response to crop diversification: A meta-analysis. Sci. Total Environ..

[24] Rosenkranz S., Wilcke W., Eisenhauer N., Oelmann Y. (2012). Net ammonification as influenced by plant diversity in experimental grasslands. Soil Biol. Biochem..

[25] Leininger S., Urich T., Schloter M., Schwark L., Qi J., Nicol G.W., Prosser J.I., Schuster S., Schleper C. (2006). Archaea predominate among ammonia-oxidizing prokaryotes in soils. Nature.

[26] Sintes E., Bergauer K., de Corte D., Yokokawa T., Herndl G.J. (2013). Archaeal amoA gene diversity points to distinct biogeography of ammonia-oxidizing Crenarchaeota in the ocean. Environ. Microbiol..

[27] Lu X., Nicol G.W., Neufeld J.D. (2018). Differential responses of soil ammonia-oxidizing Archaea and Bacteria to temperature and depth under two different land uses. Soil Biol. Biochem..

[28] Merloti L.F., Mendes L.W., Pedrinho A., De Souza L.F., Ferrari B.M., Tsai S.M. (2019). Forest-to-Agriculture Conversion in Amazon Drives Soil Microbial Communities and N-cycle. Soil Biol. Biochem..

[29] Martínez-Espinosa C., Sauvage S., Al Bitar A., Green P., Vörösmarty C., Sánchez-Pérez J. (2021). Denitrification in wetlands: A review towards a quantification at global scale. Sci. Total Environ..

[30] Coyne M.S., Lal R., Steward B.A. (2018). Denitrification in soil. Soil Nitrogen Uses and Environmental Impacts.

[31] Schulz S., Kölbl A., Ebli M., Buegger F., Schloter M., Fiedler S. (2017). Field-scale pattern of denitrifying microorganisms and N_2_O emission rates indiate a high potential for complete denitrification in an agriculturally used organic soil. Microb. Ecol..

[32] Orellana L.H., Rodriguez-R L.M., Higgins S., Chee-Sanford J.C., Sanford R.A., Ritalahti K.M., Löffler F.E., Konstantinidis K.T. (2014). Detecting Nitrous Oxide Reductase (nosZ) Genes in Soil Metagenomes: Method Development and Implications for the Nitrogen Cycle. mBio.

[33] Nugroho R.A., Röling W.F.M., Laverman A.M., Zoomer H.R., Verhoef H.A. (2005). Presence of Nitrosospira cluster 2 bacteria corresponds to N transformation rates in nine acid Scots pine forest soils. FEMS Microbiol. Ecol..

[34] Mintie A.T., Heichen R.S., Cromack K., Myrold D.D., Bottomley P.J. (2003). Ammonia-Oxidizing Bacteria along Meadow-to-Forest Transects in the Oregon Cascade Mountains. Appl. Environ. Microbiol..

[35] Avrahami S., Conrad R. (2005). Cold-temperature climate: A factor for selection of ammonia oxidizers in upland soil?. Can. J. Microbiol..

[36] Avrahami S., Liesack W., Conrad R. (2003). Effects of temperature and fertilizer on activity and community structure of soil ammonia oxidizers. Environ. Microbiol..

[37] Boyle S.A., Bottomley P.J., Myrold D.D. (2008). Community composition of ammonia oxidizing bacteria and archaea in soils under stands of red alder and Douglas fir in Oregon. Environ. Microbiol..

[38] Boyle S.A., Rich J.J., Bottomley P.J., Cromack K., Myrold D.D. (2006). Reciprocal transfer effects on denitrifying community composition and activity at forest and meadow sites in the Cascade Mountains of Oregon. Soil Biol. Biochem..

[39] Bottomley P.J., Taylor A.E., Boyle S.A., McMahon S.K., Rich J.J., Cromack Jr K., Myrold D.D. (2004). Responses of nitrification and ammonia-oxidizing bacteria to reciprocal transfers of soil between adjacent coniferous forest and meadow vegetation in the Cascade Mountains of Oregon. Microb. Ecol..

[40] Rich J.J., Heichen R.S., Bottomley P.J., Cromack Jr. K., Myrold D.D. (2003). Community Composition and Functioning of Denitrifying Bacteria from Adjacent Meadow and Forest Soils. Appl. Environ. Microbiol..

[41] Rachid C.T.C.C., Balieiro F.C., Peixoto R.S., Pinheiro Y.A.S., Piccolo M.C., Chaer G.M., Rosado A.S. (2013). Mixed Plantations Can Promote Microbial Integration and Soil Nitrate Increases with Changes in the N Cycling Genes. Soil Biol. Biochem..

[42] Balieiro F.D.C., Pereira M.G., Alves B.J.R., Resende A.S.D., Franco A.A. (2008). Soil carbon and nitrogen in pasture soil reforested with *Eucalyptus* and *Guachapele*. Rev. Bras. Ci. Solo.

[43] Forrester D.I., Bauhus J., Cowie A.L. (2005). On the success and failure of mixed-species tree plantations: Lessons learned from a model system of *Eucalyptus globulus* and *Acacia mearnsii*. For. Ecol. Manag..

[44] Khanna P.K. (1997). Comparison of growth and nutrition of young monocultures and mixed stands of *Eucalyptus globulus* and *Acacia mearnsii*. For. Ecol. Manag..

[45] Laclau J.-P., Bouillet J.-P., Gonçalves J.L.M., Silva E.V., Jourdan C., Cunha M.C.S., Moreira M.R., Saint-André L., Maquère V., Nouvellon Y. (2008). Mixed-species plantations of *Acacia mangium* and *Eucalyptus grandis* in Brazil. For. Ecol. Manag..

[46] Shibata H., Urakawa R., Toda H., Inagaki Y., Tateno R., Koba K., Nakanishi A., Fukuzawa K., Yamasaki A. (2011). Changes in nitrogen transformation in forest soil representing the climate gradient of the Japanese archipelago. J. For. Res..

[47] Jia Y., Liao Z., Chew H., Wang L., Lin B., Chen C., Lu G., Lin Z. (2020). Effect of *Pennisetum giganteum* z.x. lin mixed nitrogen-fixing bacterial fertilizer on the growth, quality, soil fertility and bacterial community of pakchoi (*Brassica chinensis* L.). PLoS ONE.

[48] Liang Y., Pan F., He X., Chen X., Su Y. (2016). Effect of vegetation types on soil arbuscular mycorrhizal fungi and nitrogen-fixing bacterial communities in a karst region. Environ. Sci. Pollut. Res..

[49] Hou G., Bi H., Wei X., Wang N., Cui Y., Zhao D., Ma X., Wang S. (2020). Optimal configuration of stand structures in a low-efficiency *Robinia pseudoacacia* forest based on a comprehensive index of soil and water conservation ecological benefits. Ecol. Indic..

[50] Meng X., Fan S., Dong L., Li K., Li X. (2023). Response of Understory Plant Diversity to Soil Physical and Chemical Properties in Urban Forests in Beijing, China. Forests.

[51] Wang N., Bi H.X., Cui Y.H., Zhao D.Y., Hou G.R., Yun H.Y., Liu Z.H., Lan D.Y., Jin C. (2022). Optimization of stand structure in *Robinia pseudoacacia* Linn. based on soil and water conservation improvement function. Ecol. Indic..

[52] Wei H., Peng C., Liu X., Chen D., Li Y., Wang M., Yang B., Song H., Li Q., Jiang L. (2018). Contrasting Soil Bacterial Community, Diversity, and Function in Two Forests in China. Front. Microbiol..

[53] Wei X., Liang W. (2019). Multifactor relationships between stand structure and soil and water conservation functions of *Robinia pseudoacacia* L. in the Loess Region. PLoS ONE.

[54] Wei X., Liang W.J. (2021). Regulation of stand density alters forest structure and soil moisture during afforestation with *Robinia pseudoacacia* L. and *Pinus tabulaeformis* Carr. on the Loess Plateau. For. Ecol. Manag..

[55] Bremner J.M., Jenkinson D.S. (1960). Determination of organic carbon in soil. J. Soil Sci..

[56] Li K., Zhao Y., Yuan X., Zhao H., Wang Z., Li S., Malhi S.S. (2012). Comparison of Factors Affecting Soil Nitrate Nitrogen and Ammonium Nitrogen Extraction. Commun. Soil Sci. Plant Anal..

[57] Page A.L., Miller R.H., Keeney D.R. (1982). Methods of Soil Analysis. Part 2: Chemical and Microbiological Properties.

[58] Cornfield A.H. (1960). Ammonia released on treating soils with N sodium hydroxide as a possible means of predicting the nitrogen-supplying power of soils. Nature.

[59] Olsen S.R., Cole C.V., Watanabe F.S., Dean L.A. (1954). Estimation of Available Phosphorus in Soils by Extraction with Sodium Bicarbonate (No. 939).

[60] Lu D., Li C., Sokolwski E., Magen H., Chen X., Wang H., Zhou J. (2017). Crop Yield and Soil Available Potassium Changes as Affected by Potassium Rate in Rice–Wheat Systems. Field Crops Res..

[61] Sparling G., West A. (1988). Modifications to the flmigation-extraction technique to permit simultaneous extraction and estimation of soil microbial c and n. Commun. Soil Sci. Plant Anal..

[62] Vance E.D., Brookes P.C., Jenkinson D.S. (1987). An extraction method for measuring soil microbial biomass carbon. Soil Biol. Biochem..

[63] Yao H., Gao Y., Nicol G.W., Campbell C.D., Prosser J.I., Zhang L., Han W., Singh B.K. (2011). Links between ammonia oxidizer community structure, abundance, and nitrification potential in acidic soils. Appl. Environ. Microbiol..

[64] Pell M., Stenberg B., Stenström J., Torstensson L. (1996). Potential Denitrification Activity Assay in Soil—With or without Chloramphenicol?. Soil Biol. Biochem..

[65] Muchane M.N., Sileshi G.W., Gripenberg S., Jonsson M., Pumarino L., Barrios E. (2020). Agroforestry boosts soil health in the humid and sub-humid tropics: A meta-analysis. Agric. Ecosyst. Environ..

[66] Van Eerd L.L., Congreves K.A., Hayes A., Verhallen A., Hooker D.C. (2014). Long-term tillage and crop rotation effects on soil quality, organic carbon, and total nitrogen. Can. J. Soil Sci..

[67] Wang Z., Liu R., Fu L., Tao S., Bao J. (2023). Effects of orchard grass on soil fertility and nutritional status of fruit trees in Korla fragrant pear orchard. Horticulturae.

[68] Cierjacks A., Kowarik I., Joshi J., Hempel S., Ristow M., Von der Lippe M., Weber E. (2013). Biological Flora of the British Isles: *Robinia pseudoacacia*. J. Ecol..

[69] De Marco A., Arena C., Giordano M., De Santo A. (2013). Impact of the invasive tree black locus ton soil properties of Mediterranean Stone pine-holm oak forests. Plant Soil.

[70] Dancer W.S., Peterson L.A., Chesters G. (1973). Ammonification and nitrification of N as influenced by soil pH and previous N treatment. Soil Sci. Soc. Am. Proc..

[71] Lehtovirta-Morley L.E., Sayavedra-Soto L.A., Gallois N., Schouten S., Stein L.Y., Prosser J.I., Nicol G.W. (2016). Identifying Potential Mechanisms Enabling Acidophily in the Ammonia-Oxidizing Archaeon “*Candidatus Nitrosotalea devanaterra*”. Appl. Environ. Microbiol..

[72] He J.-Z., Hu H.-W., Zhang L.-M. (2012). Current Insights into the Autotrophic Thaumarchaeal Ammonia Oxidation in Acidic Soils. Soil Biol. Biochem..

[73] Zhang K., Cheng X., Dang H., Ye C., Zhang Q. (2012). Soil nitrogen and denitrification potential as affected by land use and stand age following agricultural abandonment in a headwater catchment. Soil Use Manag..

[74] Wang R., Feng Q., Liao T., Zheng X., Butterbach-Bahl K., Zhang W., Jin C. (2013). Effects of nitrate concentration on the denitrification potential of a calcic cambisol and its fractions of N_2_, N_2_O and NO. Plant Soil.

[75] Li N., Shao T.Y., Zhu T.S., Long X.H., Gao X.M., Liu Z.P., Shao H.B., Rengel Z. (2018). Vegetation succession influences soil carbon sequestration in coastal alkali-saline soils in southeast China. Sci. Rep..

[76] Dickinson C.H., Pugh G.J.F. (1974). Biology of Plant Litter Decomposition.

[77] Li Q.X., Jia Z.Q., Zhu Y.J., Wang Y.S., Li H., Yang D.F., Zhao X.B. (2015). Spatial heterogeneity of soil nutrients after the establishment of *Caragana intermedia* plantation on sand dunes in alpine sandy land of the Tibet Plateau. PLoS ONE.

[78] Wu J., Wang H., Li G., Ma W., Wu J., Gong Y., Xu G. (2020). Vegetation Degradation Impacts Soil Nutrients and Enzyme Activities in Wet Meadow on the Qinghai-Tibet Plateau. Sci. Rep..

[79] Qu Z., Liu B., Ma Y., Sun H. (2020). Differences in bacterial community structure and potential functions among eucalyptus plantations with different ages and species of trees. Appl. Soil. Ecol..

[80] An S.-S., Cheng Y., Huang Y.-M., Liu D. (2013). Effects of Revegetation on Soil Microbial Biomass, Enzyme Activities, and Nutrient Cycling on the Loess Plateau in China. Restor. Ecol..

[81] Brockett B.F.T., Prescott C.E., Grayston S.J. (2012). Soil Moisture Is the Major Factor Influencing Microbial Community Structure and Enzyme Activities across Seven Biogeoclimatic Zones in Western Canada. Soil Biol. Biochem..

[82] Dai H., Dong B., Yang Z., Yuan Y., Tan Y., Huang Y., Zhang X. (2023). Mixed Plantations Improve Soil Bacterial Similarity by Reducing Heterogeneous Environmental Selection. Forests.

[83] Wang C., Zhang W., Li X., Wu J. (2022). A Global Meta-Analysis of the Impacts of Tree Plantations on Biodiversity. Glob. Ecol. Biogeogr..

[84] Hooper D.U., Bignell D.E., Brown V.K., Brussard L., Dangerfield J.M., Wall D.H., Wardle D.A., Coleman D.C., Giller K.E., Lavelle P.J.B. (2000). Interactions between Aboveground and Belowground Biodiversity in Terrestrial Ecosystems: Patterns, Mechanisms, and Feedbacks. Bioscience.

[85] Nielsen U.N., Osler G.H.R., Campbell C.D., Neilson R., Burslem D.F.R.P., Van Der Wal R. (2010). The Enigma of Soil Animal Species Diversity Revisited: The Role of Small-Scale Heterogeneity. PLoS ONE.

[86] Monard C., Vandenkoornhuyse P., Bot B., Binet F. (2011). Relationship between bacterial diversity and function under biotic control: The soil pesticide degraders as a case study. ISME J..

[87] Nioh I. (1994). Comparison of microflora of Yezo spruce (*Picea jezoensis* (Sieb. et Zucc.) Carr.) roots in the healthy and deleterious forests. Jpn. J. For. Environ..

[88] Hartman W.H., Richardson C.J., Vilgalys R., Bruland G.L. (2008). Environmental and anthropogenic controls over bacterial communities in wetland soils. Proc. Natl. Acad. Sci. USA.

[89] Cheng J.M., Zhao M.X., Cong J., Qi Q., Xiao Y., Cong W., Deng Y., Zhou J.Z., Zhang Y.G. (2020). Soil pH exerts stronger impacts than vegetation type and plant diversity on soil bacterial community composition in subtropical broad-leaved forests. Plant Soil.

[90] Siles J.A., Margesin R. (2016). Abundance and diversity of bacterial, archaeal, and fungal communities along an altitudinal gradient in alpine forest soils: What are the driving factors?. Microb. Ecol..

[91] Sariyildiz T., Anderson J., Kucuk M. (2005). Effects of tree species and topography on soil chemistry, litter quality, and decomposition in northeast turkey. Soil Biol. Biochem..

[92] Liu T., Wu X., Li H., Alharbi H., Wang J., Dang P., Chen X., Kuzyakov Y., Yan W. (2020). Soil organic matter, nitrogen and pH driven change in bacterial community following forest conversion. For. Ecol. Manag..

[93] Zhao J., Wan S., Li Z.A., Shao Y., Xu G., Liu Z., Zhou L., Fu S. (2012). Dicranopteris-dominated understory as major driver of intensive forest ecosystem in humid subtropical and tropical region. Soil Biol. Biochem..

[94] Feng Y., Grogan P., Caporaso J.G., Zhang H., Lin X., Knight R., Chu H. (2014). PH is a good predictor of the distribution of anoxygenic purple phototrophic bacteria in Arctic soils. Soil Biol. Biochem..

[95] Shen C., Xiong J., Zhang H., Feng Y., Lin X., Li X., Liang W., Chu H. (2013). Soil PH Drives the Spatial Distribution of Bacterial Communities along Elevation on Changbai Mountain. Soil Biol. Biochem..

[96] Lauber C.L., Hamady M., Knight R., Fierer N. (2009). Pyrosequencing-based assessment of soil ph as a predictor of soil bacterial community structure at the continental scale. Appl. Environ. Microbiol..

[97] Kaiser M., Kleber M., Berhe A.A. (2015). How air-drying and rewetting modify soil organic matter characteristics: An assessment to improve data interpretation and inference. Soil Biol. Biochem..

[98] Manzoni S., Schimel J.P., Porporato A. (2012). Responses of soil microbial communities to water stress: Results from a meta-analysis. Ecology.

[99] Fierer N., Jackson R.B. (2006). The diversity and biogeography of soil bacterial communities. Proc. Natl. Acad. Sci. USA.

[100] Shen C., Ni Y., Liang W., Wang J., Chu H. (2015). Distinct soil bacterial communities along a small-scale elevational gradient in alpine tundra. Front. Microbiol..

[101] Drenovsky R.E., Vo D., Graham K.J., Scow K.M. (2004). Soil Water Content and Organic Carbon Availability Are Major Determinants of Soil Microbial Community Composition. Microb. Ecol..

[102] Miranda J., Armas C., Padilla F., Pugnaire F. (2011). Climatic change and rainfall patterns: Effects on semi-arid plant communities of the Iberian Southeast. J. Arid. Environ..

[103] Butenschoen O., Scheu S., Eisenhauer N. (2011). Interactive effects of warming, soil humidity and plant diversity on litter decomposition and microbial activity. Soil Biol. Biochem..

[104] Schnürer J., Clarholm M., Boström S., Rosswall T. (1986). Effects of moisture on soil microorganisms and nematodes: A field experiment. Microb. Ecol..

[105] Sarathchandra S., Ghani A., Yeates G., Burch G., Cox N. (2001). Effect of nitrogen and phosphate fertilisers on microbial and nematode diversity in pasture soils. Soil Biol. Biochem..

[106] Gu S., Hu Q., Cheng Y., Bai L., Liu Z., Xiao W., Gong Z., Wu Y., Feng K., Deng Y. (2019). Application of organic fertilizer improves microbial community diversity and alters microbial network structure in tea (*Camellia sinensis*) plantation soils. Soil Tillage Res..

[107] Soares M., Rousk J. (2019). Microbial growth and carbon use efficiency in soil: Links to fungal-bacterial dominance, SOC-quality and stoichiometry. Soil Biol. Biochem..

[108] Peng C., Lai S., Luo X., Lu J., Huang Q., Chen W. (2016). Effects of long term rice straw application on the microbial communities of rapeseed rhizosphere in a paddyupland rotation system. Sci. Total Environ..

[109] Torres I.F., Bastida F., Hernández T., Bombach P., Richnow H.H., García C. (2014). The role of lignin and cellulose in the carbon-cycling of degraded soils under semiarid climate and their relation to microbial biomass. Soil Biol. Biochem..

[110] He Z.L., Yang X.E., Baligar V.C., Calvert D.V. (2003). Microbiological and biochemical indexing systems for assessing quality of acid soils. Adv. Agron..

[111] Wang R., Filley T.R., Xu Z., Wang X., Li M.H., Zhang Y., Luo W., Jiang Y. (2014). Coupled Response of Soil Carbon and Nitrogen Pools and Enzyme Activities to Nitrogen and Water Addition in a Semi-Arid Grassland of Inner Mongolia. Plant Soil.

[112] Deng J.J., Yin Y., Zhu W.X., Zhou Y.B. (2018). Variations in soil bacterial community diversity and structures among different revegetation types in the Baishilazi Nature Reserve. Front. Microbiol..

[113] Sun H., Terhonen E., Koskinen K., Paulin L., Kasanen R., Asiegbu F.O. (2014). Bacterial diversity and community structure along different peat soils in boreal forest. Appl. Soil Ecol..

[114] Zeng Q., Dong Y., An S. (2016). Bacterial community responses to soils along a latitudinal and vegetation gradient on the Loess Plateau, China. PLoS ONE.

[115] Zhang M., Wang W., Wang D., Heenan M., Xu Z. (2018). Short-term responses of soil nitrogen mineralization, nitrification and desnitrification to prescribed burning in a suburban forest ecosystem of subtropical Australia. Sci. Total Environ..

[116] Wei X., Bi H., Liang W., Hou G., Kong L., Zhou Q. (2018). Relationship between Soil Characteristics and Stand Structure of *Robinia pseudoacacia* L. and *Pinus tabulaeformis* Carr. Mixed Plantations in the Caijiachuan Watershed: An Application of Structural Equation Modeling. Forests.

[117] Janssen P.H. (2006). Identifying the dominant soil bacterial taxa in libraries of 16S rRNA and 16S rRNA genes. Appl. Environ. Microbiol..

[118] Zhang X.F., Zhao L., Xu S.J., Liu Y.Z., Liu H.Y., Cheng G.D. (2013). Soil moisture effect on bacterial and fungal community in Beilu River (Tibetan Plateau) permafrost soils with different vegetation types. J. Appl. Microbiol..

[119] Ivanova A.A., Beletsky A.V., Rakitin A.L., Kadnikov V.V., Philippov D.A., Mardanov A.V., Ravin N.V., Dedysh S.N. (2020). Closely Located but Totally Distinct: Highly Contrasting Prokaryotic Diversity Patterns in Raised Bogs and Eutrophic Fens. Microorganisms.

[120] Zhu P., Wang Y., Shi T., Zhang X., Huang G., Gong J. (2018). Intertidal zonation affects diversity and functional potentials of bacteria in surface sediments: A case study of the Golden Bay mangrove, China. Appl. Soil Ecol..

[121] Zhang C., Li J., Wang J., Liu G., Wang G., Guo L., Peng S. (2019). Decreased temporary turnover of bacterial communities along soil depth gradient during a 35-year grazing exclusion period in a semiarid grassland. Geoderma.

[122] Mushinski R.M., Zhou Y., Gentry T.J., Boutton T.W. (2018). Bacterial Metataxonomic Profile and Putative Functional Behavior Associated with C and N Cycle Processes Remain Altered for Decades after Forest Harvest. Soil. Biol. Biochem..

[123] Chen L., Brookes P.C., Xu J., Zhang J., Zhang C., Zhou X., Luo Y. (2016). Structural and functional differentiation of the root-associated bacterial microbiomes of perennial ryegrass. Soil Biol. Biochem..

[124] Fierer N., Bradford M.A., Jackson R.B. (2007). Toward an Ecological Classification of Soil Bacteria. Ecology.

[125] Pankratov T.A., Ivanova A.O., Dedysh S., Liesack W. (2011). Bacterial populations and environmental factors controlling cellulose degradation in an acidic Sphagnum peat. Environ. Microbiol..

[126] Radajewski S., Webster G., Reay D.S., Morris S.A., Ineson P., Nedwell D.B., Prosser J.I., Murrell J.C. (2002). Identification of active methylotroph populations in an acidic forest soil by stable-isotope probing. Microbiology.

[127] Lu S.P., Gischkat S., Reiche M., Akob D.M., Hallberg K.B., Kusel K. (2010). Ecophysiology of Fe-cycling bacteria in acidic sediments. Appl. Environ. Microb..

[128] Bryant D.A., Costas A.M.G., Maresca J.A., Chew A.G.M., Klatt C.G., Bateson M.M., Tallon L.J., Hostetler J., Nelson W.C., Heidelberg J.F. (2007). *Candidatus Chloracidobacterium thermophilum*: An aerobic phototrophic acidobacterium. Science.

[129] Ivanova A.A., Zhelezova A.D., Chernov T.I., Dedysh S.N. (2020). Linking ecology and systematics of acidobacteria: Distinct habitat preferences of the *Acidobacteriia* and *Blastocatellia* in tundra soils. PLoS ONE.

[130] Piao Z., Yang L.Z., Zhao L.P., Yin S.X. (2008). Actinobacterial community structure in soils receiving long-term organic and inorganic amendments. Appl. Environ. Microbiol..

[131] Ma B., Stirling E., Liu Y., Zhao K., Zhou J., Singh B.K., Tang C., Dahlgren R.A., Xu J. (2021). Soil Biogeochemical Cycle Couplings Inferred from a Function-Taxon Network. Research.

[132] Singh B.K., Munro S., Potts J.M., Millard P. (2007). Influence of grass species and soil type on rhizosphere microbial community structure in grassland soils. Appl. Soil Ecol..

[133] Pajares S., Bohannan B.J. (2016). Ecology of nitrogen fixing, nitrifying, and denitrifying microorganisms in tropical forest soils. Front. Microbiol..

[134] Yousuf B., Keshri J., Mishra A., Jha B. (2012). Application of targeted metagenomics to explore abundance and diversity of CO_2_-fixing bacterial community using *cbbL* gene from the rhizosphere of *Arachis hypogaea*. Gene.

[135] Maestre F.T., Delgado-Baquerizo M., Jeffries T.C., Eldridge D.J., Ochoa V., Gozalo B., Quero J.L., García-Gómez M., Gallardo A., Ulrich W. (2015). Increasing aridity reduces soil microbial diversity and abundance in global drylands. Proc. Natl. Acad. Sci. USA.

[136] Xun W.B., Wu X., Huang T., Ran W., Li D.C., Shen Q.R., Li Q., Zhang R.F. (2016). Swine manure and quicklime have different impacts on chemical properties and composition of bacterial communities of an acidic soil. Appl. Soil Ecol..

[137] Han S., Huang Q., Chen W. (2021). Partitioning nitrospira community structure and co-occurrence patterns in a long-term inorganic and organic fertilization soil. J. Soils Sed..

[138] Koch H., van Kessel M.A.H.J., Lucker S. (2019). Complete nitrification: Insights into the ecophysiology of comammox Nitrospira. Appl. Microbiol. Biotechnol..

[139] Saghaï A., Banjeree S., Degrune F., Edlinger A., García-Palacios P., Garland G., Heijden M.G.A., Herzog C., Maestre F.T., Pescador D.S. (2022). Diversity of Archaea and Niche Preferences among Putative Ammonia-oxidizing Nitrososphaeria Dominating across European Arable Soils. Environ. Microbiol..

[140] Norton J.M., Klotz M.G., Stein L.Y., Arp D.J., Bottomley P.J., Chain P.S., Hauser L.J., Land M.L., Larimer F.W., Shin M.W. (2008). Complete genome sequence of *Nitrosospira multiformis*, an ammonia-oxidizing bacterium from the soil environment. Appl. Environ. Microbiol..

[141] Castellano-Hinojosa A., González-López J., Bedmar E.J. (2018). Distinct effect of nitrogen fertilisation and soil depth on nitrous oxide emissions and nitrifiers and denitrifiers abundance. Biol. Fertil. Soils.

[142] Mushinski R.M., Gentry T.J., Dorosky R.J., Boutton T. (2017). Forest harvest intensity and soil depth alter inorganic nitrogen pool sizes and ammonia oxidizer community composition. Soil Boil. Biochem..

[143] Koops H.-P., Purkhold U., Pommerening-Röser A., Timmermann G., Wagner M. (2006). The lithoautotrophic ammonia-oxidizing bacteria. The Prokaryotes.

[144] Braker G., Zhou J., Wu L., Devol A.H., Tiedje J.M. (2000). Nitrite reductase genes (nirK and nirS) as functional markers to investigate diversity of denitrifying bacteria in Pacific Northwest marine sediment communities. Appl. Environ. Microbiol..

[145] Jones C.M., Hallin S. (2010). Ecological and evolutionary factors underlying global and local assembly of denitrifier communities. ISME J..

[146] Cavigelli M.A., Robertson G.P. (2000). The Functional Significance of Denitrifier Community Composition in a Terrestrial Ecosystem. Ecology.

[147] Liu X., Chen C., Wang W., Hughes J.M., Lewis T., Hou E., Shen J. (2015). Vertical distribution of soil denitrifying communities in a wet sclerophyll forest under long-term repeated burning. Microb. Ecol..

[148] Wang H., Li X., Li X., Li X., Wang J., Zhang H. (2017). Changes of Microbial Population and N-Cycling Function Genes with Depth in Three Chinese Paddy Soils. PLoS ONE.

[149] Butterbach-Bahl K., Baggs E.M., Dannenmann M., Kiese R., Zechmeister-Boltenstern S. (2013). Nitrous oxide emissions from soils: How well do we understand the processes and their controls?. Philos. Trans. R. Soc. B.

[150] Cheng Y., Wang J., Wang S., Zhang J., Cai Z. (2014). Effects of soil moisture on gross N transformations and N_2_O emission in acid subtropical forest soils. Biol. Fertil. Soils.

[151] Szukics U., Abell G.C.J., Hödl V., Mitter B., Sessitsch A., Hackl E., Zechmeister-Boltenstern S. (2010). Nitrifiers and denitrifiers respond rapidly to changed moisture and increasing temperature in a pristine forest soil. FEMS Microbiol. Ecol..

[152] Ribbons R.R., Levy-Booth D.J., Masse J., Grayston S.J., McDonald M.A., Vesterdal L., Prescott C.E. (2016). Linking microbial communities, functional genes and nitrogen-cycling processes in forest floors under four tree species. Soil Biol. Biochem..

[153] Zhang X., Liu S.R., Huang Y.T., Fu S.L., Wang J.X., Ming A.G., Li X.Z., Yao M.J., Li H. (2018). Tree species mixture inhibits soil organic carbon mineralization accompanied by decreased r-selected bacteria. Plant Soil.

[154] Sun X., Han X., Ping F., Zhang L., Zhang K., Chen M., Wu W. (2018). Effect of rice-straw biochar on nitrous oxide emissions from paddy soils under elevated CO_2_ and temperature. Sci. Total. Environ..

