# Supplementary material for: The Effects of Mixed Robinia pseudoacacia and Quercus variabilis Plantation on Soil Bacterial Community Structure and Nitrogen-Cycling Gene Abundance in the Southern Taihang Mountain Foothills
Source: Microorganisms. 2024 Aug 27;12(9):1773. doi: 10.3390/microorganisms12091773 (PMC11434179; doi:10.3390/microorganisms12091773)
Supplement: Supplementary file 1 [file microorganisms-12-01773-s001.zip › microorganisms-3173133-supplementary.pdf]

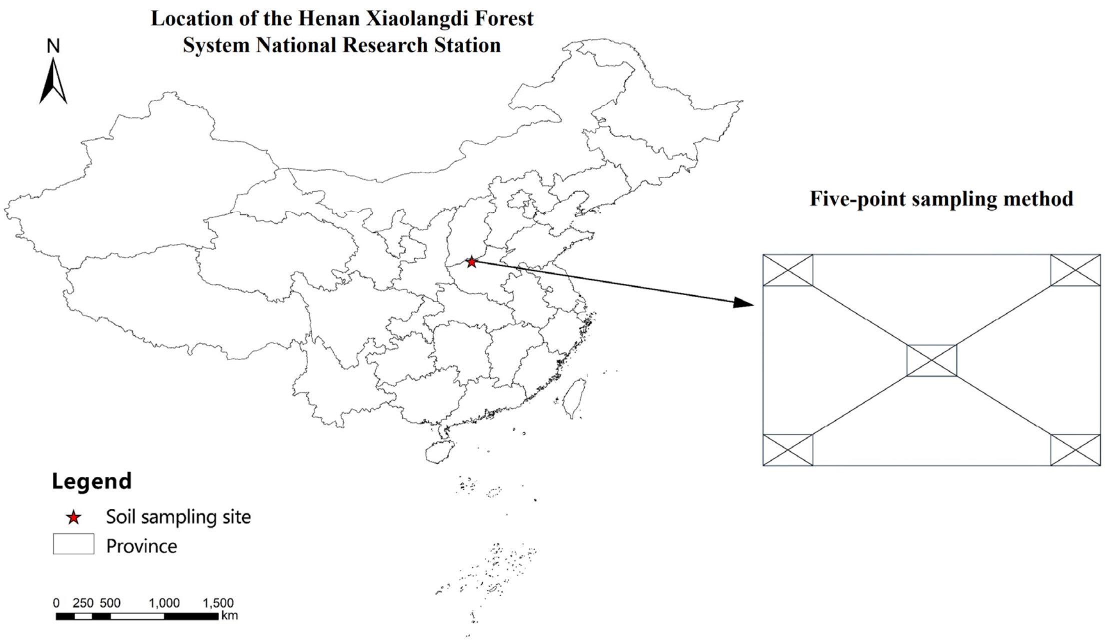

Figure S1 Soil sampling site and scheme

Table S1 Similarity analysis of bacterial communities among different samples

Sample	R value	*p* value
R5 vs R10	0.8648	0.003
Q5 vs Q10	0.5685	0.002
RQ5 vs RQ10	0.7426	0.001
R5 vs Q5	0.6704	0.003
R5 vs RQ5	0.8963	0.001
Q5 vs RQ5	0.112	0.16
R10 vs Q10	0.7926	0.002
R10 vs RQ10	0.8963	0.003
Q10 vs RQ10	0.2963	0.007

Table S2 Correlation between bacterial community structure and soil environmental factors tested by Mantel

Physicochemical properties	Bacterial community structure	
	r	*P*
pH	0.3634	0.001
SMC	0.09438	0.147
NH_4	0.01218	0.553
NO_3	0.003407	0.455
HN	0.01248	0.531
SOM	0.02666	0.645
AK	0.02	0.374
PNR	0.2259	0.019
PDR	0.06898	0.197
MBC	0.05779	0.244
MBN	0.1233	0.105
